# Interpretable Latent-Factor Machine Learning for Vapor Solubility in Amorphous Polymers

**DOI:** 10.1021/acs.jpcb.6c03323

**Published:** 2026-09-01

**Authors:** Brandon L. Foley, Cody B. Cockreham, Sylvie Aubry, Maxwell Murialdo

**Affiliations:** 4578 Lawrence Livermore National Laboratory, 7000 East Avenue, Livermore, California 94550, United States

## Abstract

Machine-learning methods are applied to predict the Henry solubility of vapors in polymers through low-rank matrix-factor models. A systematic evaluation of the predictive capability of varying model ranks shows that rank-2 models have the best predictive power without the risks of instability or overfitting for the dataset in this work. The rank-2 model predicted the Henry solubilities within a multiplicative factor of 1.5 in 95% of cases for 43 vapors in nine polymers and at six temperatures. The vapors and polymers in this study span a range of chemical characteristics, including alkanes, olefins, oxygen-containing organic molecules, and halogenated hydrocarbons. Comparison of the latent factors against the polarizability, hydrogen bonding, and volumetric character of each vapor suggests that the two latent features learned by the model are the nonspecific dispersive character and specific polar/hydrogen-bonding character of the vapor/polymer. This model enables the determination of the solvation character of additional polymers by calibrating their feature vectors on as few as four chemically distinct vapors. The feature vector for the newly calibrated polymer can then be used to predict the solubility of the remaining untested vapors within this polymer, enabling a significant reduction in the number of experiments required to complete the solubility matrix and providing an avenue for estimating the solubilities of difficult-to-measure vapor–polymer interactions. Finally, the UNIFAC 2.0 model with free-volume correction is evaluated for predicting Henry solubilities of vapors in polymers, and its performance is compared to the low-rank model derived in this work.

## Graphical abstract

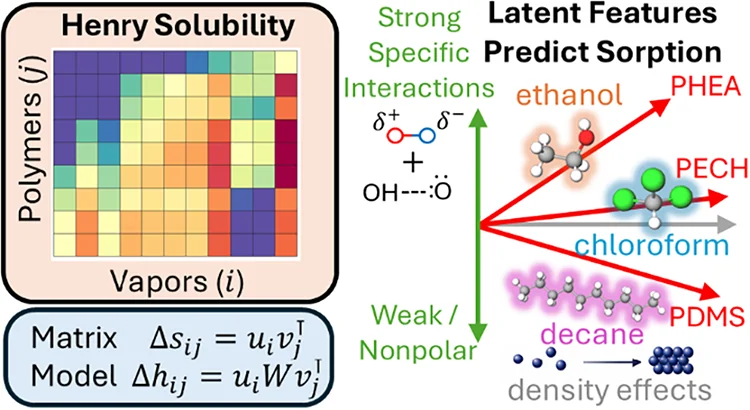

## Introduction

Predicting mixture thermodynamics remains a central challenge in physical chemistry and materials science, particularly for systems where experimental data are sparse.^1,2^ Among these, vapor sorption in polymers is especially important because it is a key determinant of barrier performance and membrane separations and is strongly coupled to long-term aging behavior.^3–5^ Classical liquid-mixture activity models^6^ and modern data-driven methods both face a common practical obstacle, namely sparse coverage of chemical space and missing interaction data. Matrix completion methods^7,8^ (MCM) offer a principled approach to learning from incomplete thermodynamic data sets by imputing missing entries in the measurement matrix. In the context of small-molecule liquids, matrix completion has been used to fill missing parameters in the UNIFAC group-interaction matrix^9,10^ (named “UNIFAC 2.0”) and to predict infinite-dilution Henry solubilities for solute–solvent pairs without invoking explicit chemical structure.^11–14^ These results suggest that matrix completion may be similarly valuable for polymer–vapor systems, where thermodynamic measurements are also sparse, incomplete, and practically significant.

Machine-learning approaches have recently been applied to related polymer–solvent thermodynamic problems. Brettman and co-workers,^15–18^ for example, developed methods to predict polymer miscibility across a range of solvents. Here, we address a related but distinct problem in a different thermodynamic regime: predicting the infinite-dilution sorption of vapors in polymers, quantified by their Henry solubilities.

Experimental sorption measurements are labor-intensive, and available literature data sets are sparse.^19–22^ Much of the vapor solubility data are spread across literature sources, while the most extensive databases, such as the Dortmund Data Bank,^20^ are paid services containing ∼492 vapors and ∼576 polymers, but only ∼1.5% of the polymer–vapor pairs are covered. It is infeasible to experimentally measure all vapor–polymer combinations of interest. Further, even this large database contains only a narrow sample of the tens of thousands of polymers that have been synthesized.^23^

The challenge is amplified by the fact that a polymer name does not uniquely specify a material. Polymers with the same repeat-unit chemistry can differ in molecular-weight distribution, crystallinity, additives, processing history, thermal history, and aging state, all of which can affect vapor sorption.^4,24–26^ Thus, the present work evaluates how a small number of measurements on the polymer or vapor of interest can be used to complete the relevant local Henry-solubility matrix through matrix completion.

The operating principle of matrix completion is that the complete matrix, ***M***, is low-rank and can be factored into two matrices ***U*** and ***V*** of rank *K*:

$$M_{m\times n}=U_{m\times K}V_{n\times K}^{†}$$

where the subscripts refer to the matrix dimensions. While ***M*** would initially appear to have *m* × *n* degrees of freedom (dof), the matrix factorization has only dof = *K*(*n* + *m* – *K*). If there are dof known elements of ***M***, then ***U*** and $V^{†}$ can be determined and the remaining elements of ***M*** can be ascertained by taking their product. The matrices ***U*** and ***V*** represent the learned latent row and column features, respectively. Each row of these matrices represents a vector of length *K* of characteristics for each vapor and polymer.

In this work, the Henry solubility, *H*_*ij*_(*T*), describes the mass of vapor *i* (*w*_*i*_) per mass of polymer *j* (*w*_*j*_) per relative pressure of vapor *i* (*P_i_*/(*P*_*i*_^sat^), where *P*_*i*_ and *P*_*i*_^sat^ are the partial and saturation pressure of vapor *i*, respectively) at temperature *T* (eq 1):

1 $$H_{\mathrm{ij}}\left(T\right)=\left(\frac{w_{i}\left(T\right)/w_{j}}{P_{i}/P_{i}^{\mathrm{sat}}\left(T\right)}\right)$$

This definition of vapor solubility is used because it emphasizes the thermodynamics of mixing and reduces the effects of molar mass and the strong vapor-sorption temperature dependence caused by the free energy of condensation, which is well described by *P*_*i*_^sat^(*T*).

The logarithm of the Henry solubility is related to the standard-state free energy of mixing vapor *i* in polymer *j* at infinite dilution (Δ*g*_*ij*_^◦^) by ln *H*_*ij*_ = −Δ*g*_*ij*_^0^/*RT* = Δ*s*_*ij*_^0^/*R* – Δ*h*_*ij*_^0^/*RT*, where Δ*s*_*ij*_^0^ and Δ*h*_*ij*_^0^ are the standard-state entropy and enthalpy of mixing vapor *i* in polymer *j* at infinite dilution and *R* is the gas constant; the standard states for this definition of solubility in eq 1 are pure liquid vapor component *i* and a polymer-phase concentration of 1 mg/g.

In the model we construct in this work, we first assume that the entropy- and enthalpy-of-mixing matrices have low effective rank, such that the dominant variation in Δ*s*_*ij*_^0^ and Δ*h*_*ij*_^0^ can be represented using a small number of latent vapor–polymer interaction modes. This low-rank assumption is implemented by assigning latent feature vectors to each vapor and polymer; thus, the entropy and enthalpy are approximated by the bilinear relationships in eq 2:

2 $$\begin{array}{l} \Delta s_{\mathrm{ij}}^{0}/R\approx u_{s,i}v_{s,j}^{†}\\ \Delta h_{\mathrm{ij}}^{0}/R\approx u_{h,i}v_{h,j}^{†} \end{array}$$

where ***u*** and ***v*** are the vapor and polymer latent feature vectors, respectively, and subscripts *s* and *h* denote the learned entropic and enthalpic features. The functional form in eq 2 is analogous to the approach taken by Damay et al.^12^ for finding infinite-dilution activity coefficients of vapors in liquid solvents.

We then make a further model-reduction assumption: the dominant latent features describing entropy and enthalpy are not independent but can be represented in a shared low-dimensional space. Specifically, we assume that the enthalpy matrix can be approximated using the entropy-derived vapor and polymer feature vectors after applying a linear transformation, ***W*** (eq 3).

3 $$\Delta h_{\mathrm{ij}}^{0}/R\approx u_{h,i}v_{h,j}^{†}\approx u_{s,i}Wv_{s,j}^{†}$$

This shared-space assumption is related in spirit to entropy-enthalpy compensation effects,^27–29^ which have been observed in many areas of kinetics and thermodynamics, where the enthalpic and entropic contributions to a chemical process are correlated. We adopt eq 3 as a testable model reduction and evaluate its adequacy quantitatively in the results below.

Combining eqs 2 and 3, the low-rank model for Henry solubility becomes (eq 4):

4 $$\ln H\left(T\right)=UV^{†}-\frac{\mathrm{UW}V^{†}}{T}$$

where ***U*** and ***V*** contain the entropy-derived vapor and polymer feature vectors, respectively.

In this study, we use matrix factorization to predict the Henry solubility for the sorption of 43 different vapors in nine different polymers from 60 to 110 °C at six different thermal conditions, utilizing data reported by Tian and Munk.^30^ The vapors and polymers vary chemically and are grouped into different chemical classifications in Tables 1 and 2. We evaluate three sampling methods—random bootstrap sampling, sampling across 13 organic-chemistry families, and sampling across four broad chemical classes defined by elemental composition and bonding—to characterize uncertainty and select the appropriate factorization rank. Specifically, these classes separate saturated hydrocarbons containing only C–H and C–C single bonds, unsaturated hydrocarbons containing C=C double bonds, oxygen-containing organics, and chlorinated hydrocarbons. This work aims to answer three key questions:

(i) What are the uncertainties in the predicted vapor sorption Henry solubilities?
(ii) How many experiments are necessary to predict solubilities for new, untrained vapors and polymers in the Henry-solubility matrix?
(iii) How do we interpret the latent features that are learned by the model?

**Table 1:** List of 43 Vapors Used in This Study, Grouped into 4 Classes and 13 Family Sub-Groupings

class	family	vapor
saturated hydrocarbons	*n*-alkanes	propane, butane, pentane, hexane, heptane, octane, nonane, decane, undecane
	cycloalkanes	cyclopentane, cyclohexane, cycloheptane, cyclooctane
unsaturated hydrocarbons	cycloalkenes	cyclohexene
	cyclodienes	cyclohexadiene
	aromatics	benzene, toluene, ethylbenzene
oxygen-containing organics	alcohols	ethanol, 1-propanol, 1-butanol, 1-pentanol
	acetates	methyl acetate, ethyl acetate, propyl acetate, *n*-butyl acetate
	ketones	acetone, methyl ethyl ketone
	cyclic ethers	tetrahydrofuran, dioxane
chlorinated hydrocarbons	monochlorinated alkanes	methyl chloride, butyl chloride, pentyl chloride, chlorohexane, chlorooctane
	polychlorinated alkanes	chloroform, methylchloroform, 1,1-dichloroethane, 1,2-dichloroethane, methylene chloride, carbon tetrachloride
	chloroalkenes	trichloroethylene
	chloroaromatics	chlorobenzene

**Table 2:** List of Nine Polymers Used in This Study, Grouped by Polymer Family

family	polymer
polyolefin	poly(ethylethylene) (PEE)
	polypropylene (PP)
polysiloxane	poly(dimethylsiloxane) (PDMS)
polyester	polycaprolactone (PCL)
polyacrylate	poly(methyl acrylate) (PMA)
	poly(ethyl acrylate) (PEA)
	poly(*n*-butyl methacrylate) (PBMA)
chlorinated polyether	polyepichlorohydrin (PECH)
hydroxyl-functionalized acrylate	poly(2-hydroxyethyl acrylate) (PHEA)

Finally, we compare the predictive capability of the low-rank model against UNIFAC 2.0—the newly modified version of UNIFAC with machine-learned interaction parameters—and demonstrate the improved performance of UNIFAC 2.0 over UNIFAC 1.0.

## Methods

### Raw Data

Specific retention volumes (*V*_r_) during inverse gas chromatography were obtained from Tian and Munk^30^ and converted to Henry solubilities using the formula

$$H=V_{r}M\frac{P^{\mathrm{sat}}\left(T\right)}{\mathrm{RT}}$$

where *M* is the molar mass and *P* ^sat^ is the vapor pressure at temperature *T*. Vapor pressures were estimated by the Antoine equation using parameters obtained from NIST.^31^

### Matrix Factorization Methods

The unknowns (***U***, ***V***, and ***W***) in eq 4 were found by using the alternating least-squares (ALS) minimization algorithm until the change in the mean-squared error between measured and predicted ln *H* over a single iteration fell below a specified tolerance. This tolerance was set at 10^–6^. Lower tolerances were tested on a small batch of simulations to ensure that no change in the results was observed.

The rank of ***W*** is a fixed hyperparameter for each model fit. The rank of ***W*** was varied in preliminary fits by representing the matrix as the product of two factors, ***A***_*W*_ ***B***_*W*_^†^, whose dimensions were *K* × rank­(***W***). For *K* = 2, negligible differences were observed between rank­(***W***) = 1 and rank­(***W***) = 2. This was repeated for *K* = 3, where rank­(***W***) > 1 had little to no improvement over rank­(***W***) = 1 for predicting vapor solubility. For this work, rank­(***W***) = 1 was chosen because it represented the simpler model with apparently equal predictive capability. The top-performing model could change with increasing data set size or types of polymers and vapors included in the data set.

### Cold-Start Row and Column Calibration

Cold-start rows (vapors) and columns (polymers) are those absent from the original training set. To incorporate these new rows and columns into the model, their feature vectors (***u***_*i*_ for vapors, ***v***_*j*_ for polymers) must be calibrated by measuring *N*_cold‑start_ Henry solubilities against vapors or polymers from the training set. The vapors or polymers used for this calibration were chosen using a regularized D-optimal selection strategy to maximize how much information is contained in the calibration set. This involves maximizing the determinant of the information matrix, max det­(***U***_X_^†^ ***U***_X_) or max det­(***V***_Y_^†^ ***V***_Y_), where ***U***_X_ and ***V***_Y_ are the subset of *N*_cold‑start_ rows of the full training matrices ***U*** and ***V*** indexed by the calibration sets *X* and Y, respectively. Cold-start vapors are then calibrated by minimizing the squared error for the new vapor using

$${û}_{i}=\underset{\begin{array}{c} u\epsilon R^{1\times K} \end{array}}{\begin{array}{c} \mathrm{argmin} \end{array}}\underset{T\epsilon T_{i}}{\sum }\underset{j\epsilon Y}{\sum }{\left(uv_{j}^{†}+\frac{\mathrm{uW}v_{j}^{†}}{T}-\ln H_{\mathrm{ij}}\left(T\right)\right)}^{2}$$

where ***û***_*i*_ is the estimated latent feature vector for cold-start vapor, *i*, and Henry solubilities, *H*_*ij*_(*T*) are measured solubilities at the temperature *T*. The sums are taken over the set of temperatures $T_{i}$ and the vapor calibration subset Y. The first two terms in the summation are ln *Ĥ*_*ij*_(*T*) for the form in eq 4 as an example, but these terms are replaced with the appropriate functional form as necessary. An analogous optimization criterion is used to calibrate cold-start polymer feature vectors, ***v̂***_*j*_.

Double cold-start data are the pairwise vapor–polymer Henry solubilities where neither the polymer nor the vapor was in the original training set. Instead, the feature vectors for each were calibrated on a small, informed sampling of the training vapors and polymers as described above. As such, the double cold-start predictions represent the strongest test of the low-rank models.

### Pairwise Bootstrap Simulations

To reduce the influence of random chance on the best-fit model rank, we employed pairwise bootstrap simulations over 10^4^ samples and iterated over each sample for each model rank, *K*, from one to five. The number of training rows/columns used to calibrate cold-start columns/rows was fixed for each *K* at *N*_cold‑start_ = 5 to ensure fair comparisons. The RMSE of the untrained data sets was calculated as a function of *K* for the untrained cold-start data. The same random seed was used for each pairwise simulation. Initial guesses were also preallocated into a large matrix, such that the initial guess for shared rows and columns of ***U***, ***W***, and ***V*** would be identical for varying *K*.

### Interpretation of Latent Factors Using Solvent Characteristics

Two important latent factors are identified in this data set, and we assess their chemical interpretation using Abraham solvation descriptors: excess molar refraction (*E*), McGowan characteristic volume (*V*), dipolarity/polarizability (*S*), hydrogen bond acidity (*A*), and hydrogen bond basicity (*B*). Parameters were obtained from the UFZ-LSER online database^32^ and summarized in Section S3. Eight vapors were omitted from the analysis because their parameters were missing from this database (cyclohexadiene, methyl chloride, methylene chloride, butyl chloride, pentyl chloride, chlorohexane, chlorooctane, methylchloroform). Relative coefficient contributions of the solvation parameters were calculated by mean-centering and normalizing by the standard deviation. These variables are denoted with a tilde. In this analysis, Ṽ was replaced with $\tilde{V/\mathrm{MW}}$ because molecular weight contributions are included in the mass-based Henry solubility. The relative contribution of each variable for describing factor 1 was estimated by solving:

$$F_{1}=\mathrm{X̃}\beta +\gamma 1$$

where ***F***_1_ is the vector of factor 1, **X̃** is the matrix of solvation parameters, and γ is a constant intercept and **1** is a vector of ones. Because each variable in **X̃** has mean zero and unit variance, the relative importance of each variable *j* for describing factor 1 is estimated by χ_*j*_.

$$\chi_{j}=\frac{\beta_{j}^{2}}{\sum \beta_{j}^{2}}$$

Larger values of χ_*j*_ indicate greater contributions to the regression model for factor 1.

### UNIFAC + FV and UNIFAC 2.0 + FV Calculations

The UNIFAC with free-volume (FV) corrections was employed using the model described by Oishi and Prausnitz.^33^ UNIFAC 2.0 used the same equations but different interaction parameters. Parameter details are provided in Section S4.

## Results and Discussion

### Determining Model Rank

A key hyperparameter in matrix factorization is the number of latent factors, or rank, used in the model. If this number is too small, the model may fail to capture important structures in the data; if it is too large, the model may overfit, become unstable, and lose predictive accuracy. To determine the number of features of the model for the vapor solubility in polymers, we compared the RMSE for predicting vapor solubility in cold-start polymers for 10^4^ paired bootstrap simulations with varying model ranks. The pairing is done to ensure that the same random bootstrap is evaluated and compared with varying numbers of matrix features.

The probability that increasing the number of features improved the predictive RMSE for a given random bootstrap was compared as a function of the model rank, as shown in Figure 1. Including one feature in the model was better than zero features in 100% of the bootstrap simulations because the rank zero model has no predictive power. Increasing the number of features to two improved the predictive capability of the model for 99% of the bootstraps. Subsequent increases to three and four features improved predictive RMSE in 67 and 59% of bootstraps, respectively, while five features decreased the predictive RMSE with respect to the four-feature models in more than 50% of the bootstrapped samples. These results provide strong evidence that there are at least two latent features in the solubility data, while increasing the complexity of the model to three or more features does not improve the predictive capabilities by much more than a coin flip. These observations are further supported by comparison of the RMSE, which decreases significantly when moving to the rank-2 model, but further increases to model rank result in modest decreases in the RMSE. Thus, we chose *K* = 2 for the matrix model, hereafter referred to as the “rank-2 model.”

**Figure 1:**
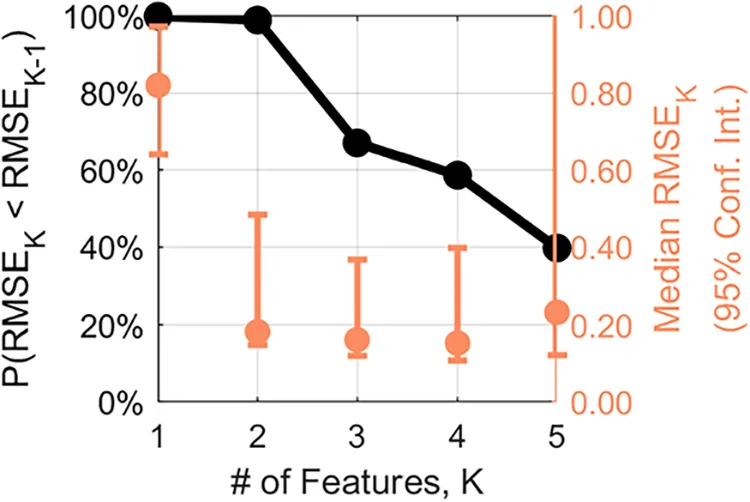
(Left axis) The probability that *K* features improved the predictive capability of the model over *K*-1 features as measured by RMSE. *N*_cold‑start_ = 5 for estimating the cold-start polymer and vapor features. (right axis) The median RMSE as a function of the number of features, with 95% confidence intervals shown as error bars.

### Data-Driven Support for Entropy–Enthalpy Compensation

In the preceding discussion, it was assumed that the latent features describing absorption entropies and enthalpies in polymers are shared, giving the model form in eq 4. However, this is justified primarily by the empirical observation of compensation effects in sorption systems. We can validate whether these compensation effects appear in our vapor–polymer absorption data by fitting the more generalized model given in eq 5:

5 $$\ln H\left(T\right)=U_{s}V_{s}^{†}-\frac{U_{h}V_{h}^{†}}{T}$$

where ***U***_*s*_ and ***V***_*s*_ are the learned entropic latent features for the vapors and polymers, while ***U***_*h*_ and ***V***_*h*_ are those for enthalpy. This functional form allows the model to learn distinct latent features for entropic and enthalpic contributions if they are present in the experimental data.

After fitting the model in eq 5 to the entire data set, we construct the orthonormal basis using singular value decomposition:

$$U_{s}V_{s}^{†}=U_{s}^{\prime }S_{s}{V_{s}^{\prime }}^{†}$$

$$U_{h}V_{h}^{†}=U_{h}^{\prime }S_{h}{V_{h}^{\prime }}^{†}$$

Principal angle analysis of ***U***_*s*_^*′*^, ***U***_*h*_^′^ and ***V***_*s*_^′^, ***V***_*h*_^′^ reveals the angles between the principal axes for the learned latent features of entropy and enthalpy, where angles of zero indicate that the latent features of entropy and enthalpy lie in the same space, while angles of 90° would indicate that they reside in independent, orthogonal spaces. The principal angles between these spaces were ***θ***_*u*_ = [7°, 33°] and ***θ***_*v*_ = [4°, 34°], suggesting that the principal axes lie in similar spaces, with one principal axis shared strongly, supporting the hypothesis that entropy and enthalpy share latent features.

Next, we evaluated whether the slight difference in the entropic and enthalpic feature spaces increased the predictive power of the model. We found that eq 5 had lower predictive RMSE 67% of the time, with median RMSE decreasing from 0.206 to 0.200 when compared to the shared latent space model in eq 4. This suggests that while the latent features of the entropic and enthalpic spaces may differ slightly, allowing them to have distinct latent spaces does not increase the predictive capability of the model for new cold-start vapors or polymers for the data in this study. However, in other systems there could be stronger evidence to support the more generalized model form in eq 5.

### Uncertainty Quantification of the Rank-2 Model

For the two-feature model, the Henry solubility is predicted within a factor of 2 for polymer cold-start, vapor cold-start, and double cold-start scenarios with ∼95% prediction coverage, as illustrated by the residual probability density functions (PDFs) in Figure 2. The cold-start residual PDFs (Figure 2a–c) follow Laplace-like distributions (heavy tails), while the training data is closer to a Gaussian distribution. For the data in Figure 2, bootstrapped sampling was used to select the training vapors and polymers, resulting in an average of 63.2% of each being selected for training, which corresponds to 5.7 polymers and 27.2 vapors on average. The features for cold-start polymers (and vapors) were then estimated by training on the two vapors (and polymers) whose feature vectors were most different and provided maximal information about the system, as described in the Methods section. The resulting predictions for the remaining Henry solubilities over 10,000 bootstrapped samples are those reported in Figure 2.

**Figure 2:**
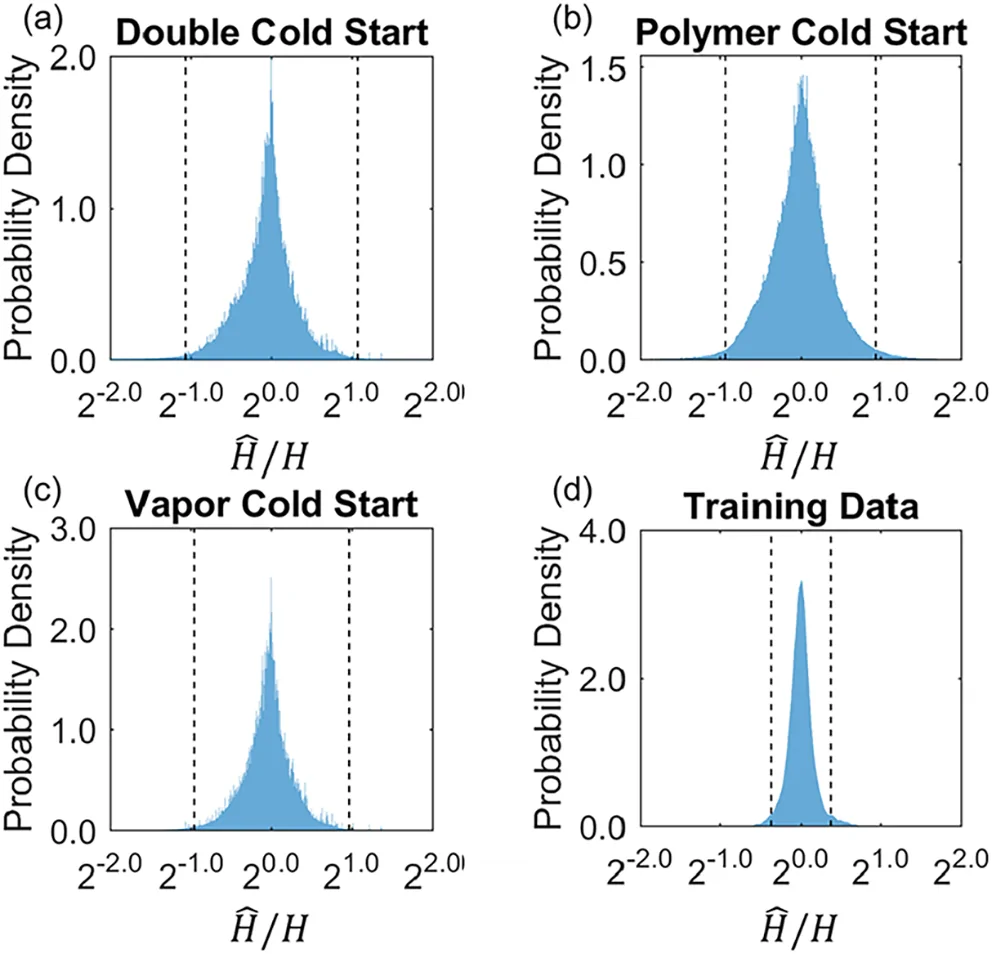
PDFs of the ratio of predicted Henry solubility (*Ĥ*) to the measured Henry solubility (*H*) with bootstrapped training samples. Dashed lines are the 95% prediction intervals for the rank-2 model. Two calibration vapors and polymers were used to estimate the cold-start polymer and vapor features, respectively. (a) Double cold-start predictions. (b) Polymer cold-start predictions. (c) Vapor cold-start predictions. (d) Training fits.

The residual PDFs in Figure 2 are for random bootstrap samples, where the molecules and polymers that represent obvious extremes based on their chemical structures may be excluded from the training sample. However, in practice, vapors and polymers would not be randomly sampled from all possible candidates; they would be selected to provide broad coverage of the relevant chemical space. Thus, random sampling may result in larger uncertainties than expected from chemically guided sampling methods. We achieve this using the vapor and polymer classifications in Tables 1 and 2 as a qualitative guide; however, other quantitative characteristics, such as Hansen solubility parameters or Abraham solvation parameters, could be used to assist in classifying molecules. We emphasize that any classifications should not be treated definitively; instead, these are hypotheses that should be evaluated.

In addition, the analysis in Figure 2 used only a minimum of two pairwise interactions for cold-start calibrations. However, more calibration data are generally expected to lead to decreased prediction uncertainties. To explore the effect of sampling method and cold-start calibration count, we reproduced the results in Figure 2 after replacing bootstrapped sampling with random sampling of one vapor from each of the 13 families in Table 1 and repeated this analysis by sampling from the four classes in Table 1. The polymers were sampled by selecting the PHEA and PCL polymers and one polyolefin/polysiloxane and one polyacrylate/polyester. The prediction uncertainty was assessed for each sample as a function of the number of calibration points for new cold-start vapors and polymers. The 95% multiplicative prediction intervals are reported in Figure 3.

**Figure 3:**
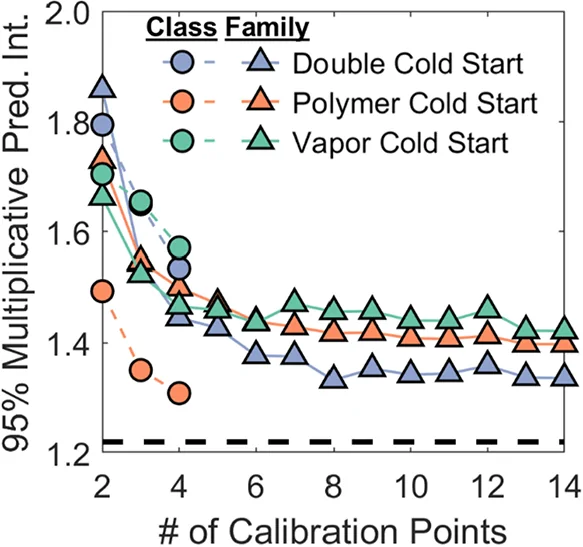
95% multiplicative prediction interval as a function of the number of calibration points used for cold-start polymers (orange), cold-start vapors (green), and the double cold-start (blue), with 100 random samples selecting one vapor from each of 13 molecular families (▲), or each of 4 classes (●) (see Table 1) for the training set. The *x*-axis is the number of vapors (and polymers) used to calibrate the features for newly added polymers (and vapors). These represent the number of pairwise interactions that are studied for each additional vapor or polymer to predict the activity coefficient within a factor given by the *y*-axis with 95% prediction coverage. There is little effect of sampling across broadly defined chemical classes versus sampling across more narrowly defined chemical families of the vapors for the training set. Four polymers were randomly selected for each training sample: one polyolefin/siloxane, one polyacrylate/ester, polyepichlorohydrin, and poly­(2-hydroxyethyl acrylate). The black dashed line indicates the 95% in-sample reconstruction-error threshold for the rank-2 model fit to the complete data set and serves as an empirical lower-bound benchmark for cold-start prediction error.

Figure 3 shows that sampling across families in Table 1 (13 vapors in the training set, ▲) gives comparable predictive power to sampling across classes (four vapors in the training set, ●). In both cases, double cold-start, cold-start vapor, and cold-start polymer predictions have 95% multiplicative prediction intervals of ∼1.5 when using four calibration points, decreasing only modestly to ∼1.4 as additional calibration points are added. For a new polymer within the chemical space represented by the data set, this suggests that measuring four vapor sorption interactions is sufficient to predict the remaining 39 vapor–polymer solubilities within a factor of 1.5 with 95% prediction coverage. The median multiplicative residual for cold-start and double cold-start prediction is <1.2 in all cases (see Figure S1). Additionally, we find that a training set comprising two vapors and two polymers with distinct chemical functionalities is sufficient to predict solubilities within a factor of 2 with 95% prediction coverage in this data set (Figure S2). For comparison, the black dashed line in Figure 3 marks the 95th-percentile multiplicative reconstruction error for the rank-2 model fits to the complete data set.

Using the qualitative chemical-based sampling method, the evidence for higher rank models becomes slightly more robust, with each sequential increase in model rank leading to ∼70% probability of improving the double cold-start RMSE, up to the rank-6 model (see Figure S3). For predicting liquid-mixture activity coefficients, models as high as rank-9 have been employed.^12^ The existence of additional latent factors is further supported by a sequential decrease in the 95% prediction interval by ∼0.05 for each increase in model rank (Figure S3). However, given the small data set size and given that the much simpler rank-2 model captures most of the predictive performance, we use the rank-2 model for the remainder of the analysis. In addition, while higher rank models may offer higher predictive capabilities, there is a trade-off with a higher number of calibration experiments required for adding new columns to the solubility matrix.

Table 3 benchmarks the calibrated rank-2 model against two group-contribution approaches for predicting Henry solubilities in polymers: UNIFAC with free-volume corrections (UNIFAC + FV)^33^ and UNIFAC 2.0 ^10^ with free-volume corrections (UNIFAC 2.0 + FV). To our knowledge, this is the first evaluation of UNIFAC 2.0 for vapor sorption in polymers. Histograms of the residuals are provided in Section S3. This comparison between the rank-2 model and UNIFAC is not strictly one-to-one because the UNIFAC-based models are purely predictive once parametrized, whereas the rank-2 model requires a small number of calibration measurements to determine the latent features of each new vapor or polymer. However, the results show that this modest experimental investment substantially improves the predictive accuracy. The original UNIFAC + FV model gives a 95% multiplicative prediction factor of 3.70, while the machine-learned interaction parameters in UNIFAC 2.0 reduce this value to 2.99. In comparison, the rank-2 model gives a 95% multiplicative prediction factor of 1.48 after calibration. Thus, for applications where a few targeted Henry-solubility measurements are feasible, the rank-2 approach provides a practical compromise between full experimental characterization and purely predictive group-contribution methods. Further, not all functional groups are available in UNIFAC or UNIFAC 2.0 models, such as those in methyl chloride and trichloroethylene, whose Henry solubilities could not be predicted for any polymer by either of the UNIFAC models.

**Table 3:** Comparison of Multiplicative Prediction Intervals for Rank-2 Model and UNIFAC + FV Models

model	95% pred. int.	50% pred. int.
Rank-2 model (*N*_cold‑start_ = 4)	1.48×	1.11×
UNIFAC + FV	3.70×	1.43×
UNIFAC 2.0 + FV	2.99×	1.37×
UNIFAC 2.0 + FV Exclusive	1.67×	1.25×

For the 20 PDMS-vapor systems that could not be evaluated with the original UNIFAC + FV model because of missing interaction parameters, UNIFAC 2.0 + FV enables their estimation by providing a more complete set of group-interaction parameters. These predictions are reported in Table 3 as “UNIFAC 2.0 + FV Exclusive” and give a 95% multiplicative prediction factor of 1.67. This subset provides preliminary evidence that the updated UNIFAC 2.0 parameters can extend free-volume-corrected UNIFAC predictions to vapor–polymer systems that were inaccessible using the original parametrization. Nevertheless, across the broader data set considered here, the calibrated rank-2 model gives the narrowest prediction interval, suggesting that limited calibration measurements can provide substantial value when higher predictive precision is needed.

In the preceding section, we analyzed the predictive performance of the low-rank model for vapor–polymer solubilities. We found that a rank-2 model trained on as few as four chemically disparate polymers and vapors, respectively, can predict solubilities for systems outside the training set within a factor of 1.5, with 95% prediction coverage. In the following section, we will explore the learned latent features of the low-rank model to interpret the factors driving sorption in this data set and understand why limited data provides strong predictive power.

### Interpretation and Insights from the Learned Latent Features

The rank-2 model was trained on the entire data set to obtain the latent features for each polymer and vapor in the data set. The model parameter fits and residuals are reported in Section S2. The model parameters are the learned latent features in the form of a two-element vector for each vapor (***u***_*i*_) and polymer (***v***_*j*_) in the study. These latent feature vectors are gauge invariant, meaning the best fits are nonunique and can be rotated or rescaled with no impact on the model predictions; thus, we use singular value decomposition (SVD) on the feature matrix product ***UV*** ^†^ to obtain a canonical orthonormal basis for interpreting the latent geometry. Using SVD, we obtain the orthonormal matrices ***U*** ^*’*^ and ***V*** ^*’*^ for row and column features, respectively (see eq 6). The matrix ***S*** scales the principal axes and is apportioned equally to the row and column features to obtain U_*_ and V_*_.

6 $$\begin{array}{l} UV^{†}=U^{\prime }S{V^{\prime }}^{†}=\left(U^{\prime }\sqrt{S}\right){\left(V^{\prime }\sqrt{S}\right)}^{†}=U_{*}V_{*}^{†}\\ U_{*}=U^{\prime }\sqrt{S}\\ V_{*}=V^{\prime }\sqrt{S} \end{array}$$

The rank-2 SVD biplot in Figure 4 visualizes the latent vapor–polymer geometry with vapors represented as points and polymers as vectors from the origin. The entropic contribution to solubility is given by the vapor–polymer dot product; therefore, vapors that project strongly along a polymer vector have more favorable predicted sorption with that polymer, whereas vapors projecting in the opposite direction have less favorable predicted sorption. The resulting geometry separates hydrophilic poly­(2-hydroxyethyl acrylate), which aligns with alcohols, from hydrophobic polymers such as poly­(ethylethylene), polypropylene, and poly­(dimethyl­siloxane), which align more closely with *n*-alkanes.

**Figure 4:**
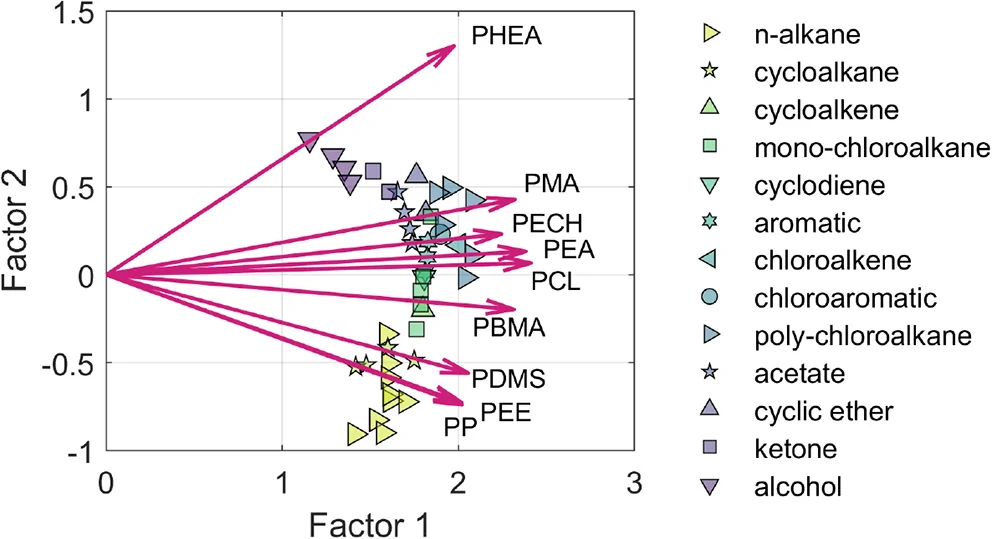
SVD biplot of learned latent vapor features, u_*,*i*_, (points) and polymer features, v_*,*j*_, (vectors) in the rank-2 matrix model. Polymer vectors are labeled by the abbreviations in Table 2. Point shapes correspond to the chemical family and coloring corresponds to the mean family angle formed with *n*-alkanes. Vapor–polymer pairs along similar axes and larger distances from the origin have greater absorption. Vectors are scaled down by a factor of 1.7 for visual clarity. Axes are square to preserve angles.

The rank-2 model predicts unknown vapor–polymer solubilities by estimating low-dimensional vapor and polymer coordinates from known pairwise data. Because the factorization is fixed by SVD conventions, the latent axes are reproducible for this data set, apart from arbitrary sign flips of each factor. Factor 1 has a uniform sign and a spread of approximately 1 across both the vapor and polymer coordinates. Thus, factor 1 does not encode a selectivity reversal among the sampled chemistries but instead contributes a sorption term shared across the vapor–polymer pairs in this data set. We interpret this behavior as consistent with broadly favorable, nonspecific sorption contributions. Although factor 1 appears visually narrow, it contributes approximately a 10^2^-fold variation to the predicted pairwise solubilities.

In contrast to factor 1, factor 2 changes sign across both the vapors and polymers, allowing the model to represent specific chemical interactions. With the sign convention used here, vapors with positive factor 2 values, such as alcohols, interact favorably with polymers that also have positive factor 2 values, such as poly­(2-hydroxyethyl acrylate). However, this same latent feature also explains why alcohols have unfavorable predicted sorption contributions in polymers with negative factor 2 values such as poly­(dimethylsiloxane). Conversely, *n*-alkanes lie on the opposite side of this factor and are favored by the hydrophobic polymers. Thus, factor 2 is consistent with the interpretation that it quantifies the specific interaction contrast between polar or hydrogen-bonding-compatible sorption and nonpolar dispersive sorption.

We further support these chemical interpretations of the latent factors by assessing their relationships with the Abraham solvation descriptors in Figure 5. Tildes denote mean-centered, unit-variance variables. Factor 2 is approximately proportional to *S̃* + *Ã* + *B̃*, which combines vapor dipolarity/polarizability with hydrogen-bond donor and acceptor character. This is consistent with the above interpretation that factor 2 captures a specific chemical interaction involving polar, noncovalent, electrostatic interactions.

**Figure 5:**
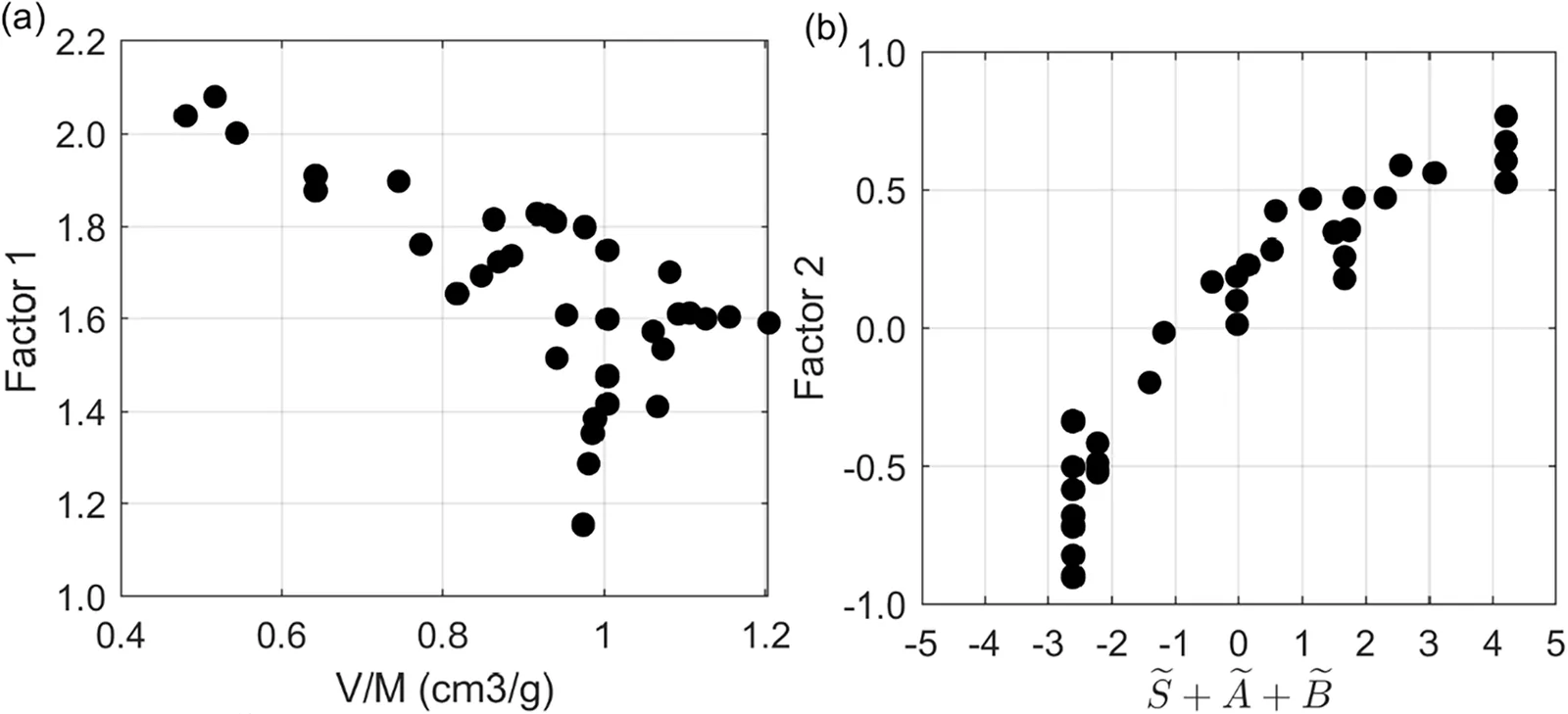
Comparison of latent factors to solvation characteristics. (a) Factor 1 versus the ratio of the McGowan Volume (*V*) to the molecular weight (*M*). In Abraham solvation parameters, *V* is typically reported scaled down by a factor of 100; here, *V* is rescaled to have units of cm^3^/mol. (b) Factor 2 versus the sum of the mean-centered and normalized values for the dipolarizability, acidic hydrogen bonding, and basic hydrogen-bonding characteristics.

Factor 1 is primarily associated with the ratio of the McGowan volume to the molar mass, *V*/*M*. This likely arises because the use of a mass-based sorbed phase standard state introduces an explicit molecular-weight dependence, reducing the extent to which molecular-size effects appear independently through *V*. An analysis of the Abraham descriptors gave the relative coefficient contributions reported in Table 4, with *V*/*M* showing the strongest alignment with factor 1, with small contributions from the H-bonding acidity and basicity descriptors *A* and *B*, and little contribution from dipolarity/polarizability descriptor, *S*, or excess molar refraction, *E*. Factor 1 therefore appears to correlate primarily with molecular size relative to mass with some secondary hydrogen-bonding corrections. This is consistent with factor 1 primarily reflecting nonspecific, nonpolar interactions.

**Table 4:** Relative Descriptor Contributions to Factor 1^a^

solvation descriptor	relative coefficient contribution for factor 1
*E*	0.00
*S*	0.07
*A*	0.27
*B*	0.17
*V*/*M*	0.48

a Calculated as normalized squared regression coefficients for standardized descriptors. Larger values indicate stronger alignment between descriptor and factor 1.

The comparatively subtle interpretation of factor 1 reflects the reference states used to define the Henry solubility. Normalization by relative pressure removes the pure-component vaporization contribution, while the mass-based sorbent concentration removes some of the size-correlated contribution. Consequently, much of the generic condensability and molecular-size variation that would be prominent for a gas-phase, molar-basis partition coefficient is already removed or embedded in the response variable, and factor 1 instead captures residual molecular volume and nonspecific interaction behavior relative to that baseline.

The latent factors may correlate with solvation descriptors while also representing variation that is not fully described by those tabulated parameters. The observed correlations therefore support the chemical relevance of the latent space without implying that the Abraham descriptors provide a complete mechanistic interpretation. We next examined the relative positions of vapors and polymers within this space, using their angles relative to decane to identify both broad chemical trends and subtler molecular distinctions.

Figures 6 and 7 provide one-dimensional summaries of the same rank-2 latent space shown in Figure 4 by using the angle with decane as a common metric for decane-like sorption behavior. This representation improves readability by allowing individual vapors and polymers to be labeled while preserving the main geometric relationships shown in the biplot. The angles between u_*,*i*_ and u_*,decane_ are reported for all 43 vapors in Figure 6 and color-coded by chemical family. These angles distinguish broad differences between vapor families while also resolving systematic variations among molecules within each family.

**Figure 6:**
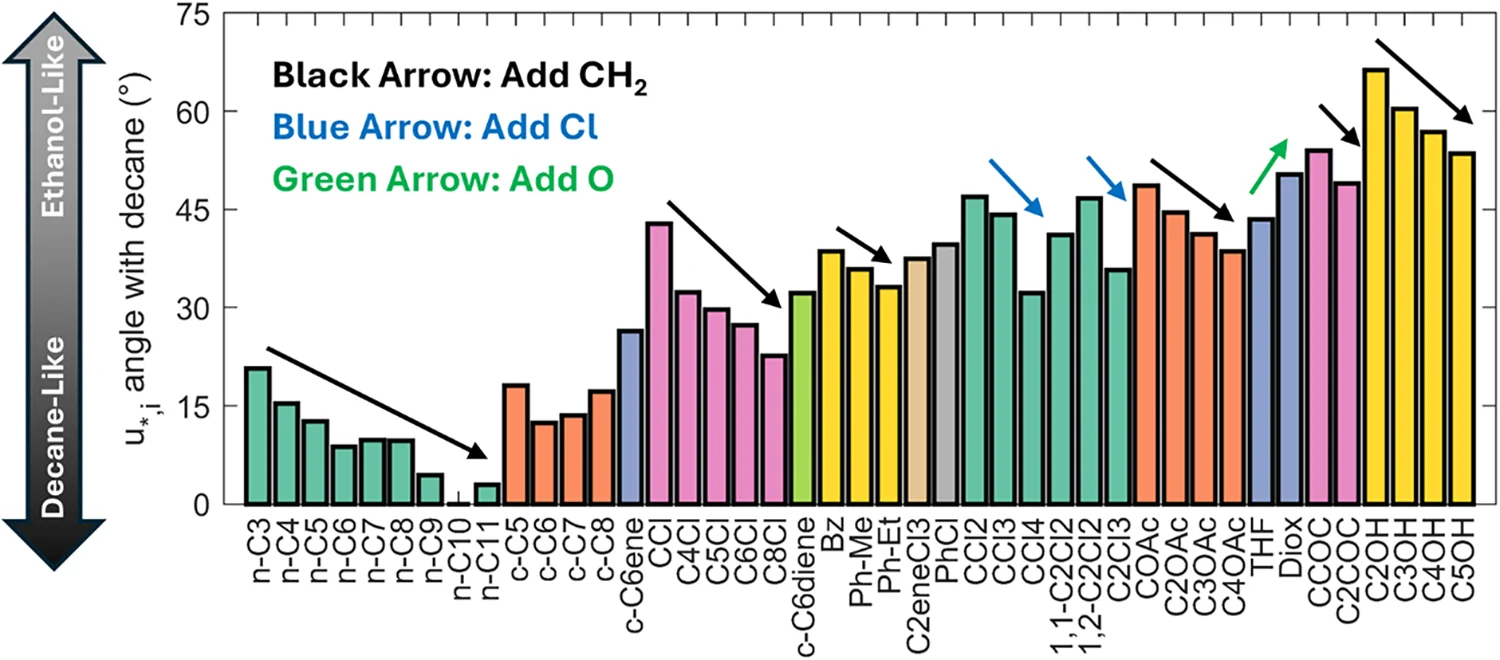
Angles between u_*,i_ for vapor *i* and u_*,decane_, a measure of how decane-like each vapor behaves. Bars are grouped by chemical family. Trends are indicated with color-coded arrows: (black) add CH_2_ group, (blue) add Cl group, (green) add O. Adding CH_2_ groups consistently decreased the angle of the feature vector with decane. Adding Cl had the same effect, while adding oxygen increased angles with decane.

**Figure 7:**
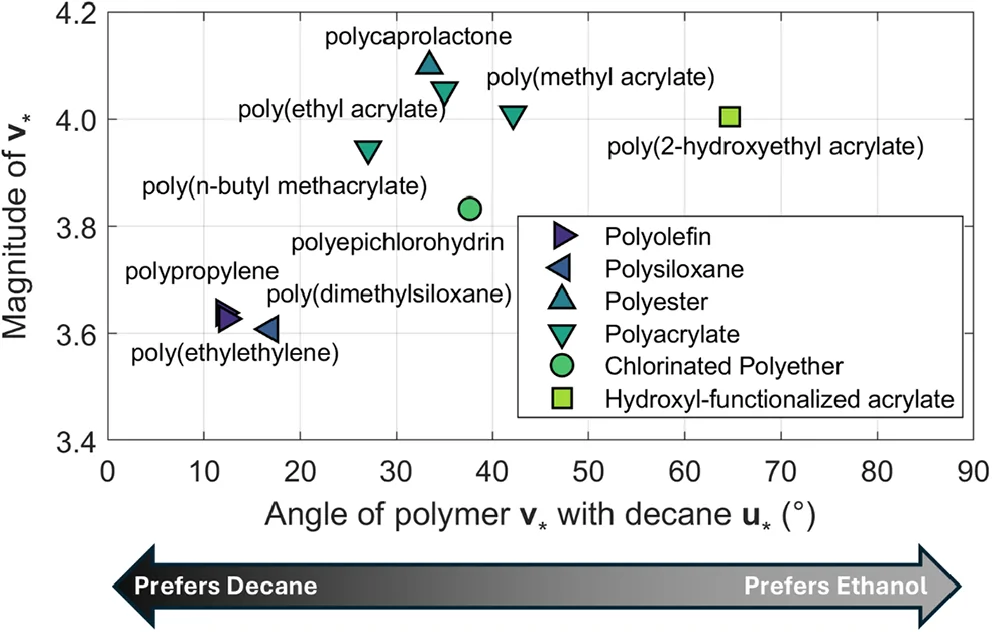
Polymer feature vectors expressed as the magnitude of v_*,*j*_ and the angle between v_*,*j*_ for the polymer and u_*,*i*_ for decane.

In general, for the acetates, alcohols, aromatics, ketones, and chlorinated alkanes, additional CH_2_ groups (increasing the carbon chain length) decrease the angle between the vapor feature vector and the decane feature vector, implying the sorption behavior of the vapor becomes more “decane-like.” Increasing the number of oxygen atoms in the cyclic ethers increases the angle with decane, likely due to increased hydrogen-bonding character, and among *n*-alkanes, vapors with carbon numbers closest to that of decane have the smallest angles with decane. Further, increasing the Cl substitution of hydrocarbons generally decreased the angle with decane, while increasing the degree of unsaturation from *n*-hexane, to cyclohexane, to cyclohexadiene, to benzene, monotonically increased the angle with decane. These subtleties are well-captured by the rank-2 matrix model and are strong evidence that the rank-2 model fits real, latent features in the sorption data.

This analysis is repeated for the polymers, with the magnitudes of v_*,*j*_ and the angles between v_*,j_ and u_*,decane_ reported in Figure 7. Again, polymers in the same or similar families have similar learned features, with hydrophobic polymers (polyolefins, polysiloxane) exhibiting small angles with decane, while hydrophilic poly­(2-hydroxyethyl acrylate) forms the largest angle with decane. We also observe that the polyesters match closely with the polyacrylate, which makes chemical sense because the only distinction between these two polymers is whether the ester group is part of the polymer backbone or the side chain. Within the polyacrylates, the angle with decane decreases with an increase in the CH_2_ content in the polymer, following the trend observed among the vapors.

## Conclusion

Machine learning is a powerful tool for building low-rank matrix models for vapor solubility in amorphous polymers. These models predict vapor sorption within a multiplicative factor of ∼1.5 for cold-start vapors and polymers at 95% prediction coverage. New vapors and polymers added to the model need to be within the span of the chemical space formed by the training vapors and polymers for accurate predictive power. In this work, we used qualitative chemical classes as proxies for selecting the initial vapors and polymers, but this process can be guided by molecular descriptors such as Hansen solubility parameters, polarizability, hydrogen-bonding descriptors, or other application-specific features. For adding new vapors and polymers to the rank-*K* model, we propose measuring 2×*K* to 4×*K* vapor–polymer combinations selected to probe chemically distinct regions of the training space and then using leave-one-out fitting to test the model’s predictive capability. If the predictive performance is poor, this may indicate that the new vapor or polymer exhibits solubility behavior outside the span of the selected training data. In that case, the model may require additional latent dimensions, a different chemically relevant training subset, or additional measurements to capture the missing solubility variation. The matrix factorization model is also useful for predicting the Henry solubilities for difficult-to-measure pairwise interactions. A particular Henry solubility may be challenging to measure due to slow diffusion, low solubilities, or safety concerns, but the polymer and vapor of interest may be well-characterized by other Henry solubilities that are easier to measure experimentally.

The row and column feature vectors of the rank-2 matrix model cluster similar molecules together, and the features track well with dispersive interactions and polar/H-bonding interactions, providing support that the latent features that are learned by the model represent real chemical characteristics of the vapors and polymers. Future work will explore the efficacy of low-rank models for describing vapor sorption thermodynamics for a wider range of vapors and polymers, including semicrystalline polymers, copolymers, glassy polymers, and filled polymers.

## Supplementary Material



## Notes

Figures showing the 50% prediction interval of the rank-2 model; probability density histograms of the residuals for a 2 × 2 training set grid using the rank-2 model; figure showing probability of RMSE improvement when using chemically informed sampling methods; results of the rank-2 model fit to the entire data set; analysis of the residual for each pairwise interaction for both the rank-2 model and individual van’t Hoff fits to each Henry solubility as a function of temperature; tables detailing the fitted parameters of the rank-2 model; compiled Abraham solvation parameters for each vapor in this study; analysis of UNIFAC + FV and UNIFAC 2.0 + FV performance for predicting Henry solubilities in this data set; tabulated parameters and functional group counts used for each vapor and polymer in the UNIFAC and UNIFAC 2.0 calculations (PDF)
