# Supplementary material for: Interpretable Latent-Factor Machine Learning for Vapor Solubility in Amorphous Polymers
Source: J Phys Chem B. 2026 Sep 1;130(37):9678–87. doi: 10.1021/acs.jpcb.6c03323 (PMC13592020; doi:10.1021/acs.jpcb.6c03323)
Supplement: Supplementary file 1 [file jp6c03323_si_001.pdf]

Supporting Information for “Interpretable Latent-Factor Machine Learning for Vapor Solubility in Amorphous Polymers”

Brandon Foley*,†,[1], Cody Cockreham[1], Sylvie Aubry[1], and Maxwell Murialdo[1]

*Corresponding author email: foley26@llnl.gov

[1]Lawrence Livermore National Laboratory, 7000 East Avenue, Livermore, CA 94550

This work was performed under the auspices of the U.S. Department of Energy by Lawrence Livermore National Laboratory under Contract DE-AC52-07NA27344.

S1. Supplemental Figures

Figure S1 shows the 50% multiplicative prediction interval as a function of the number of calibration points used in the rank-2 model for polymer cold-start, vapor cold-start, and double cold-start predictions. The training sets were samples using chemical segregation by molecular family (triangles) or molecular class (circles) from Table 1 in the main text. The median error between prediction and measurement was < ~15% in all cases, with little change in increasing number of calibration points or sampling method.

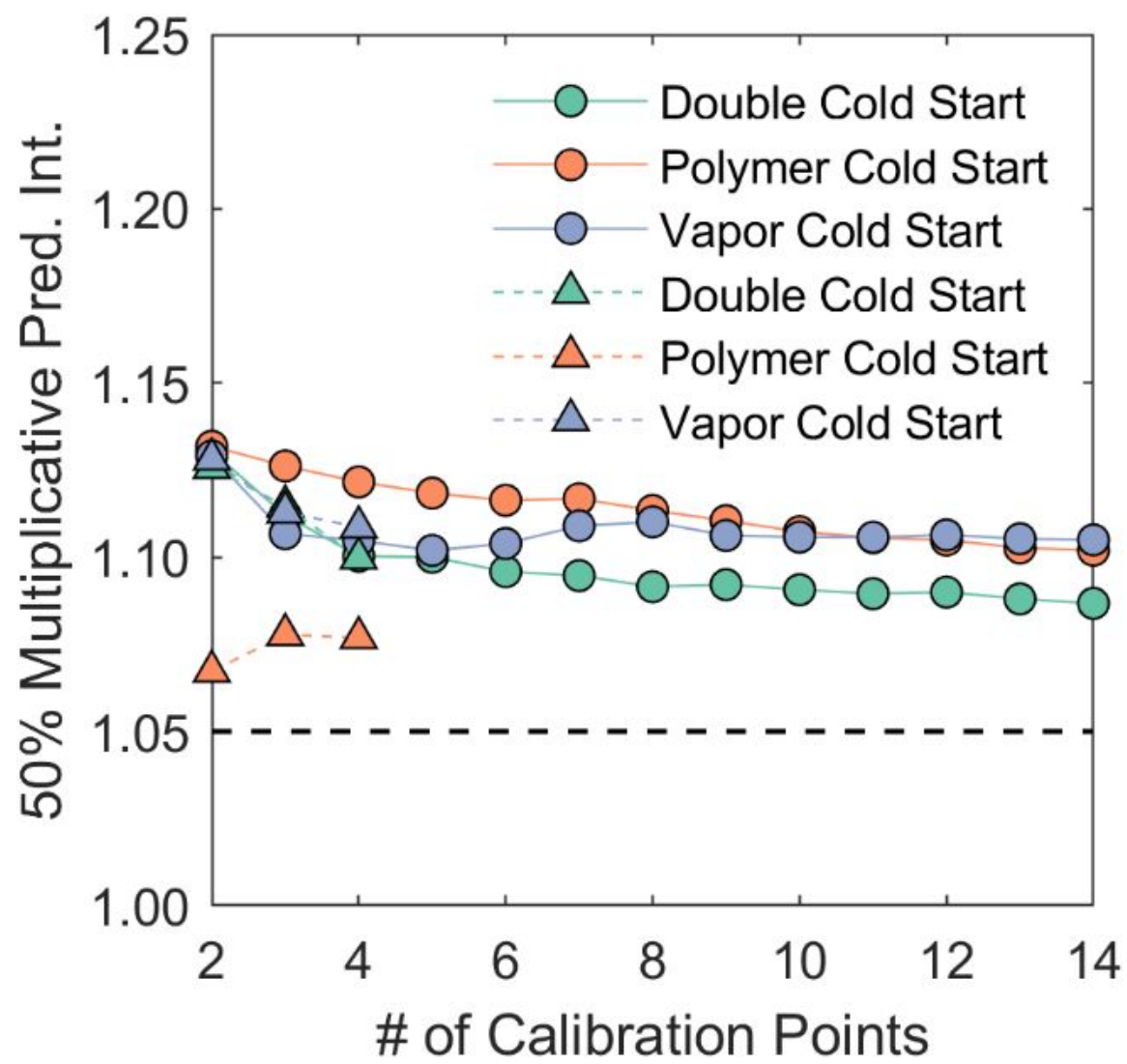

Figure S1. The 50% multiplicative prediction interval as a function of the number of calibration points used for cold-start polymers (orange), cold-start vapors (green), and the double cold-start (blue). Triangles are the prediction intervals over 100 random samples selecting one vapor from each molecular family, while circles are the prediction intervals when selecting one vapor from each of the five broad molecular classes for the training set. The x-axis is the number of vapors (and polymers) used to calibrate the features for newly added polymers (and vapors). These represent the number of pairwise interactions that are studied for each additional vapor or polymer to predict the activity coefficient within a factor given by the y-axis with 50% prediction coverage. There is little impact of sampling by chemical family or class. Four polymers were randomly selected for each training sample: one polyolefin/siloxane, one polyacrylate/ester, polyepichlorohydrin, and poly(2-hydroxyethyl acrylate).

Figure S2 shows the probability density of the residuals for vapor cold-start, polymer cold-start, and double cold-start predictions for the rank-2 model using only decane and ethanol as the training vapors and poly(ethyl ethylene) and poly(2-hydroxyethyl acetate) as the training polymer. By training the rank-2 model on only four vapor-polymer solubility pairs, the model predicts experimental solubilities within a factor of ~2 with 95% prediction coverage in all cold-start datasets.

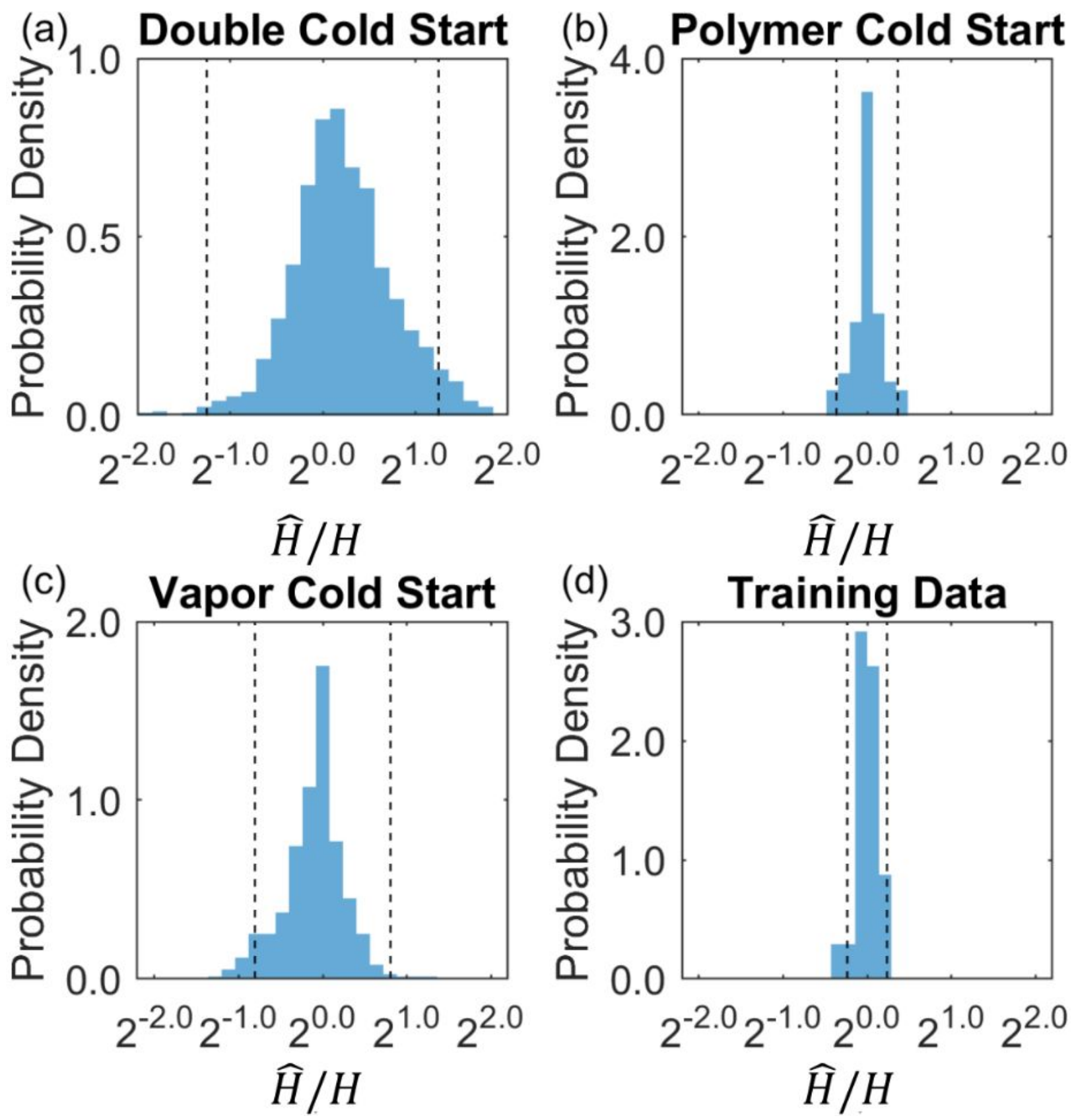

Figure S2. Histograms of the ratio of predicted Henry's constant ($\hat{H}$) to the measured Henry's constant (Henry's constant (H) with two training vapors (decane and ethanol) and two training polymers (poly(ethylethylene) and poly(2-hydroxyethyl acrylate) selected based on chemical intuition. Dashed lines are the 95% prediction intervals for $K = 2$ features. (a) Double cold-start predictions. (b) Polymer cold start predictions. (c) Vapor cold start predictions. (d) Training fits.

Sampling by chemical families rather than by random bootstraps reveals the potential presence of higher rank features in the solubility data. Each subsequent feature added to the matrix factorization model decreased the RMSE in ~70% the random samples for K > 2. Additional features also narrow the residual probability density function, with the 95% prediction interval decreasing from ~1.5 at K = 2 to ~1.3 at K = 6. This is suggestive that these are real latent features that can be learned by the model that were missed by random bootstrapped sampling due to insufficient number of unique polymers sampled. This was remedied by guiding the sampling based on molecular structure. The gains obtained for the increased model complexity were insufficient for us to prefer higher rank models in this work due to the small dataset size. However, larger datasets or applications where an improvement from ~1.5 to ~1.3 error is highly valued would support using higher rank models for estimating the vapor solubilities.

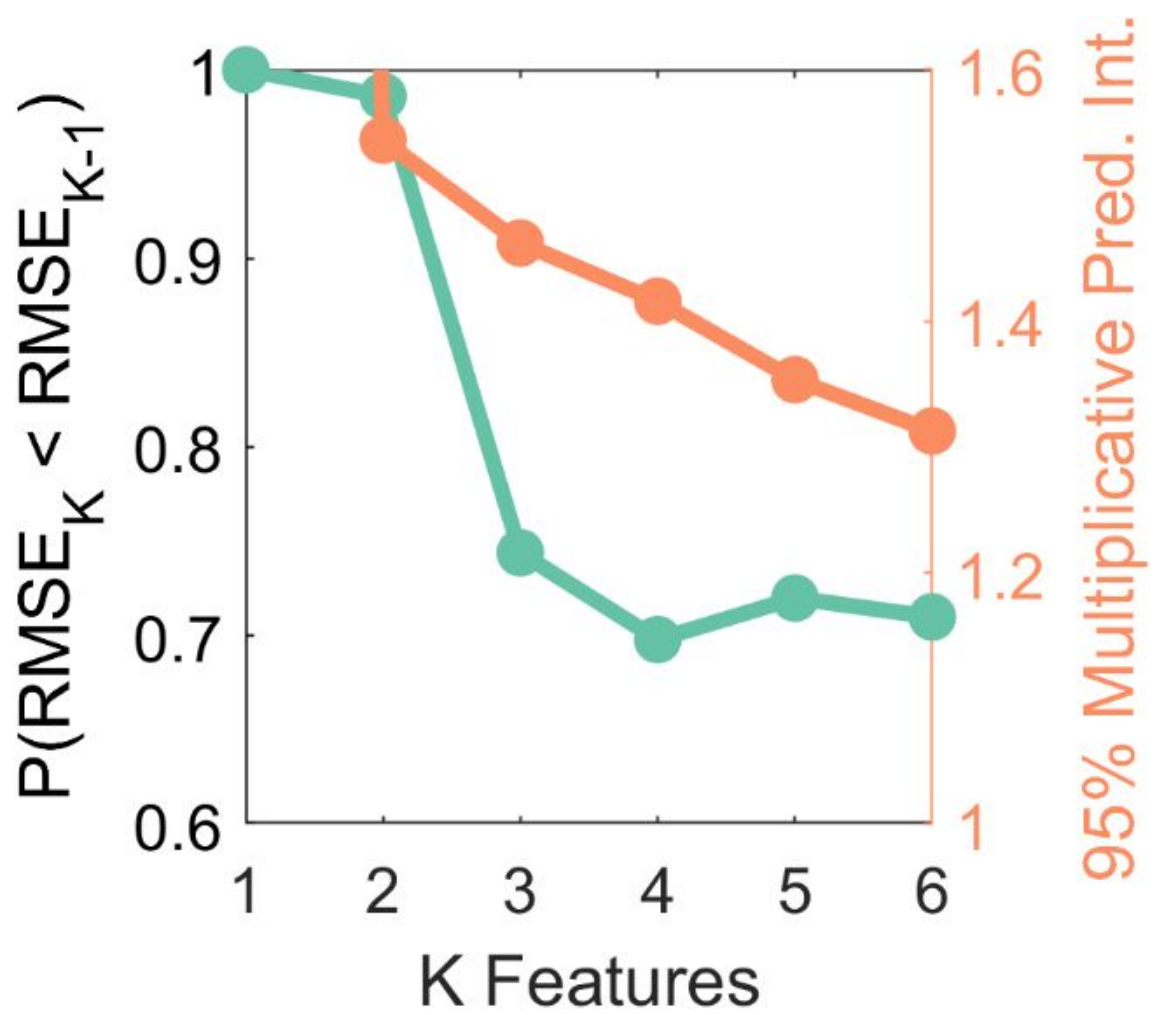

Figure S3. Left axis (green): The probability that increasing the number of matrix features improved the RMSE of the double cold-start predictions. Right axis (orange): The 95% multiplicative prediction interval for the solubility as a function of matrix features. K = 1 has a 95% predication interval of ~8 (not shown). Six polymers and six vapors were randomly sampled without replacement and ensuring that at least one polymer and vapor was selected from each chemical family in Table 1 and Table 2, with polyolefins and polysiloxanes, polyesters and polyacrylates, and chlorinated polyether and hydroxyl-functionalized acrylate grouped together. Cold-start training set is six for all training sets.

S2. Fitting result of the rank-2 model

The rank-2 model residuals to the entire vapor-polymer dataset is provided in Figure S4, with (a) reporting the parity plot between the model fit and the experimental value and (b) reporting the probability density function (PDF) of the residuals. For the complete data fit, 95% of the in-sample fitted values are within a factor of approximately 1.22 of the measured values (dashed lines in Figure S4b).

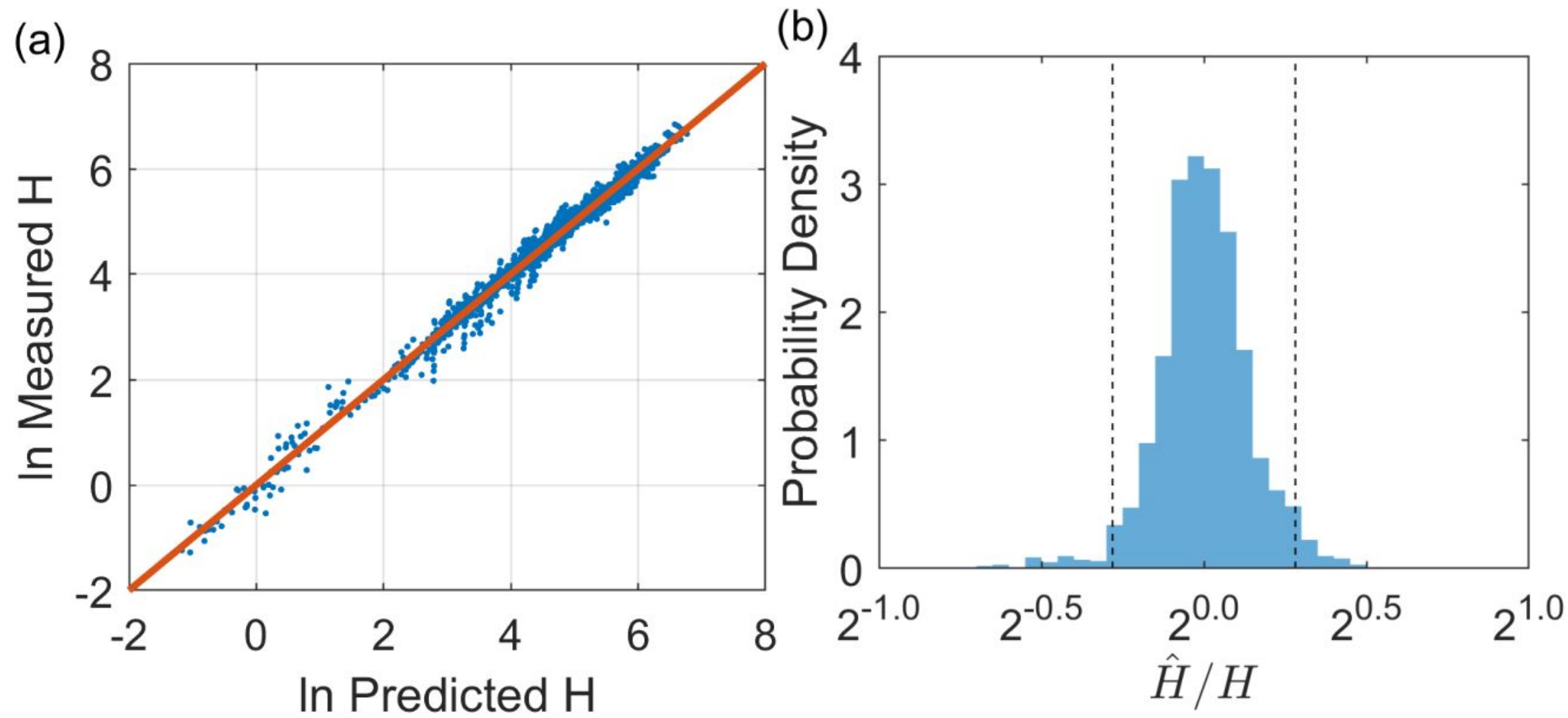

Figure S4. (a) Parity plot and (b) residual PDF for $K = 2$ model including all data in training set. 95% of the model fits fall within a factor of 1.22 of the measured value (vertical dashed lines).

Inspection of the median multiplicative residual for each vapor-polymer combination revealed the worst fit vapors were the alcohols on any polymer besides the most polar polymers, PHEA and PMA, as shown in Figure S5. Propane was poorly fit compared to the other vapors; inspection of the experimental data suggests that the propane measurements were noisy, likely due to the much lower retention volumes for this high saturation pressure gas. Based on Figure S5 alone, it would be easy to conclude that the low-rank model performs most poorly on vapor-polymer combinations with opposing dipolar/non-polar chemical behaviors.

However, In Figure S6 we show the median multiplicative errors for the van't Hoff fits to each individual vapor-polymer combination. These fits represent the lower limit of the low-rank model performance because van't Hoff behavior is presumed. High errors in the van't Hoff fits are likely reflective of noisy data points, and these are highest for many of the same vapor-polymer combinations that the low-rank model performs most poorly on. This noisy data not only limits the performance of the low-rank model but also introduces error in the estimation of the parameters themselves, leading to further decreased predictive power. Despite this, the median multiplicative error across all pairwise

combinations never exceeded 1.68, with most median multiplicative errors falling below 1.1.

Median Multiplicative Error Between $H_{fit}(T)$ and $H(T)$

	PEE	PP	PDMS	PCL	PECH	PHEA	PMA	PEA	PBMA
propane	1.07	1.08	1.53	1.24	1.68	1.47	1.31	1.22	1.04
butane	1.03	1.12	1.34	1.03	1.14		1.20	1.03	1.06
pentane	1.06	1.09	1.25	1.05	1.12	1.26	1.16	1.10	1.06
hexane	1.16	1.18	1.10	1.08	1.03	1.32	1.26	1.13	1.03
heptane	1.06	1.04	1.10	1.04	1.07	1.30	1.10	1.04	1.03
octane	1.07	1.06	1.07	1.07	1.06	1.23	1.06	1.03	1.04
nonane	1.09	1.06	1.03	1.10	1.06	1.25	1.09	1.05	1.14
decane	1.12	1.03	1.08	1.16	1.07	1.06	1.09	1.06	1.18
undecane	1.03	1.03	1.08	1.12	1.10	1.09	1.08	1.01	1.20
cyclopentane	1.03	1.01	1.08	1.02	1.03	1.29	1.10	1.01	1.08
cyclohexane	1.04	1.05	1.04	1.03	1.09	1.37	1.21	1.06	1.04
cycloheptane	1.05	1.03	1.14	1.04	1.07	1.26	1.14	1.03	1.04
cyclooctane	1.10	1.08	1.20	1.04	1.10	1.32	1.04	1.10	1.03
cyclohexene	1.12	1.07	1.02	1.12	1.05	1.27	1.08		1.10
cyclohexadiene	1.11	1.07	1.02	1.10	1.05	1.09	1.04		1.09
benzene	1.08	1.03	1.09	1.09	1.11	1.03	1.06	1.08	1.05
toluene	1.06	1.05	1.17	1.08	1.16	1.03	1.06	1.05	1.03
ethylbenzene	1.04	1.06	1.22	1.07	1.16	1.07	1.06	1.05	1.05
methyl chloride	1.10	1.07	1.20	1.19	1.15	1.23	1.02		1.17
methylene chloride	1.05	1.06	1.02	1.11	1.16	1.09	1.09	1.06	1.04
chloroform	1.07	1.02	1.11	1.12	1.31	1.08	1.12	1.13	1.12
carbon tetrachloride	1.13	1.06	1.02	1.07	1.08	1.19	1.07	1.07	1.08
butyl chloride	1.01	1.02	1.00	1.06	1.05	1.08	1.06	1.05	1.03
pentyl chloride	1.00	1.01	1.06	1.08	1.08	1.03	1.07	1.06	1.09
chlorohexane	1.02	1.01	1.13	1.07	1.10	1.04	1.05	1.04	1.08
chlorooctane	1.05	1.02	1.25	1.14	1.12	1.14	1.03	1.03	1.19
1,1-dichloroethane	1.03	1.04	1.03	1.08	1.08	1.03	1.10	1.06	1.04
1,2-dichloroethane	1.05	1.07	1.17	1.08	1.07	1.09	1.12	1.08	1.05
methylchloroform	1.06	1.05	1.05	1.06	1.05	1.23	1.07	1.03	1.08
trichloroethylene	1.15	1.13	1.08	1.12	1.18	1.16	1.10	1.08	1.06
chlorobenzene	1.08	1.06	1.28	1.08	1.07	1.04	1.08	1.08	1.06
acetone	1.14	1.13	1.15	1.08	1.26	1.08	1.02	1.03	1.09
methyl ethyl ketone	1.08	1.06	1.08	1.10	1.28	1.06	1.04	1.06	1.05
tetrahydrofuran	1.20	1.16	1.14	1.30	1.15	1.34	1.24	1.27	1.23
dioxane	1.10	1.08	1.03	1.12	1.21	1.10	1.09	1.17	1.18
methyl acetate	1.08	1.10	1.21	1.10	1.13	1.03	1.05	1.07	1.16
ethyl acetate	1.06	1.08	1.18	1.11	1.16	1.03	1.06	1.05	1.12
propyl acetate	1.07	1.07	1.11	1.10	1.20	1.07	1.06	1.06	1.09
n-butyl acetate	1.08	1.07	1.05	1.06	1.23	1.10	1.06	1.08	1.04
ethanol	1.31	1.29	1.33	1.30	1.41	1.05	1.08	1.29	1.29
1-propanol	1.29	1.28	1.26	1.28	1.38	1.05	1.07	1.23	1.33
1-butanol	1.27	1.24	1.21	1.30	1.30	1.09	1.08	1.21	1.38
1-pentanol	1.26	1.28	1.20	1.39	1.32	1.09	1.07		1.49

Figure S5. Heat map of the median multiplicative error between the model fit and the measured value for the Henry solubility at all temperatures for each vapor-polymer combination.

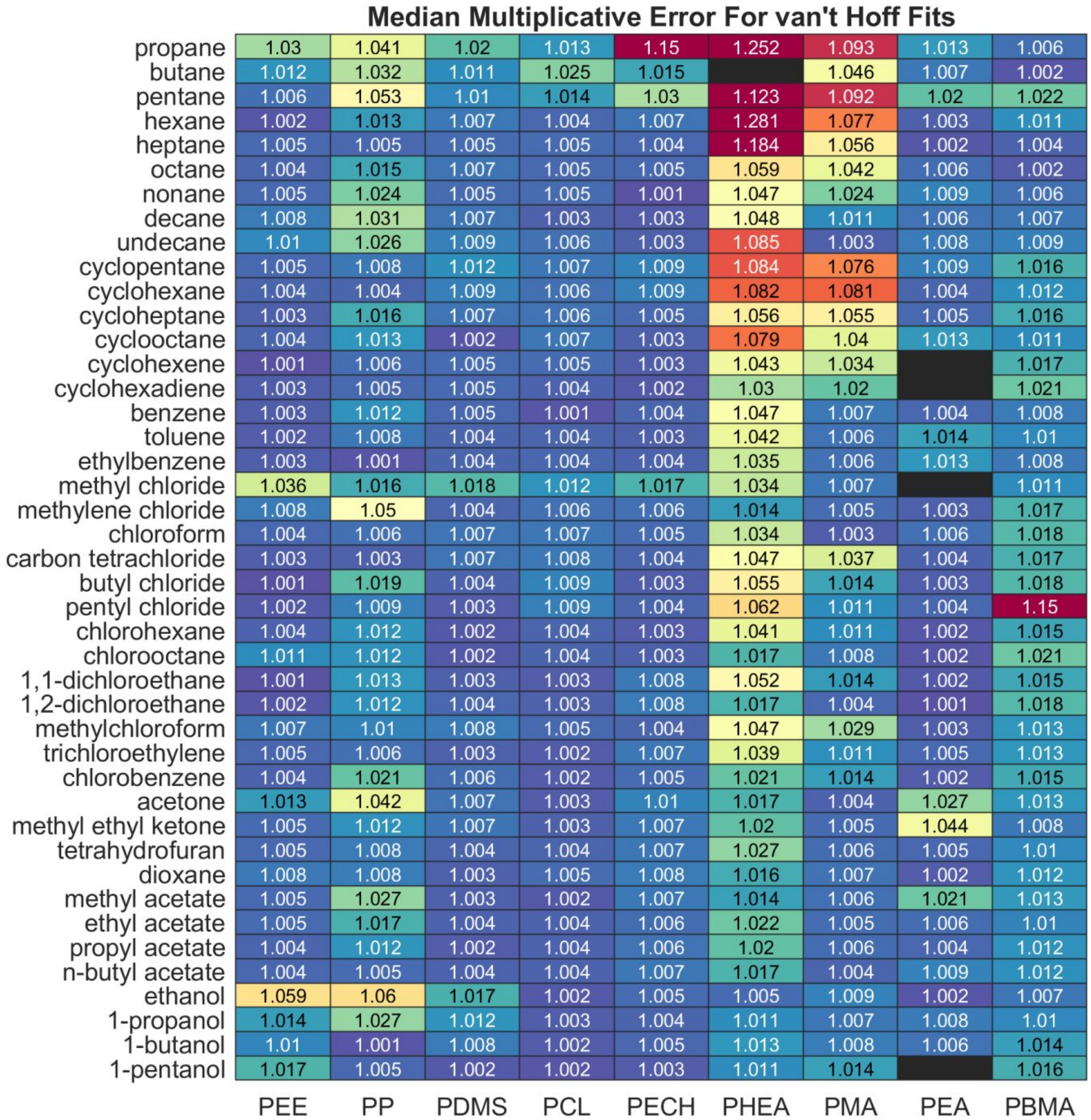

Figure S6. Median multiplicative errors for van’t Hoff fits to each pairwise vapor-polymer combination of Henry solubilities as a function of temperature.

The fitted values for this rank-2 model are provided in Tables S1–S4. These results are provided in the form obtained by singular value decomposition of $UV^{\intercal}$, such that the predicted Henry solubility for any vapor-polymer pair i-j at temperature, T, is given by:

$$\ln \hat{H}_{ij} = u_i' \boldsymbol{S} v_j'^{\intercal} + \frac{u_i' \boldsymbol{W} v_j'^{\intercal}}{T}$$

Table S1. Vapor U' parameters from rank-2 model fit.

Vapor	u'1	u'2	Vapor	u'1	u'2
propane	-0.14178	0.107932	butyl chloride	-0.1604965	0.00353323
butane	-0.14293	0.160701	pentyl chloride	-0.1593379	0.02954339
pentane	-0.14257	0.187106	chlorohexane	-0.1590555	0.05349932
hexane	-0.14367	0.229447	chlorooctane	-0.1568504	0.09950652
heptane	-0.14349	0.217445	1,1-dichloroethane	-0.170155	-0.0911223
octane	-0.15163	0.23095	1,2-dichloroethane	-0.1671978	-0.1496353
nonane	-0.13669	0.264077	methylchloroform	-0.1851548	-0.0355467
decane	-0.12577	0.289925	trichloroethylene	-0.178376	-0.0539939
undecane	-0.14014	0.287553	chlorobenzene	-0.169038	-0.073919
cyclopentane	-0.14251	0.132953	acetone	-0.1349422	-0.1890686
cyclohexane	-0.12627	0.16759	methyl ethyl ketone	-0.1433115	-0.1510116
cycloheptane	-0.13156	0.164305	tetrahydrofuran	-0.1617417	-0.1112482
cyclooctane	-0.15587	0.155883	dioxane	-0.1570089	-0.1799898
cyclohexene	-0.16025	0.063484	methyl acetate	-0.1474436	-0.1508987
cyclohexadiene	-0.16085	0.004629	ethyl acetate	-0.1509132	-0.1140973
benzene	-0.1628	-0.06038	propyl acetate	-0.1536125	-0.0833127
toluene	-0.16245	-0.03241	n-butyl acetate	-0.1548169	-0.0578808
ethylbenzene	-0.16136	-0.00457	ethanol	-0.1029356	-0.2463224
methyl chloride	-0.16428	-0.10579	1-propanol	-0.1146743	-0.2166612
methylene chloride	-0.17393	-0.15846	1-butanol	-0.1205523	-0.194134
chloroform	-0.18545	-0.13579	1-pentanol	-0.1234217	-0.1687698
carbon tetrachloride	-0.18174	0.005132			

Table S2. Polymer V' parameters from the rank-2 model fit.

Polymer	v'1	v'2
PEE	-0.30613	0.401772
PP	-0.30566	0.395197
PDMS	-0.31148	0.304012
PCL	-0.36545	-0.03618
PECH	-0.34007	-0.12606
PHEA	-0.2992	-0.70839
PMA	-0.35172	-0.23296
PEA	-0.36101	-0.07226
PBMA	-0.35083	0.107113

Table S3. W matrix from rank-2 model fit.

W	
-11781.1	-4763.91
2713.197	1097.133

Table S4. S matrix from rank-2 model fit.

S	
125.9367	0
0	9.746609

S3. Abraham Solvation Parameters from the UFZ-LSER Database

Abraham solvation parameters retrieved form the UFZ-LSER online database[1] are reported in Table SX below. When they were multiple sources with differing parameters for a given variable, preference was given to UFZ-LSER sources.

Table S5. Summary of Abraham solvation descriptors for each vapor obtained from the UFZ-LSER database.[1]

Vapor	E	S	A	B	V
propane	0	0	0	0	0.5313
butane	0	0	0	0	0.6722
pentane	0	0	0	0	0.8131
hexane	0	0	0	0	0.954
heptane	0	0	0	0	1.0949
octane	0	0	0	0	1.2358
nonane	0	0	0	0	1.3767
decane	0	0	0	0	1.5176
undecane	0	0	0	0	1.6585
cyclopentane	0.26	0.1	0	0	0.7045
cyclohexane	0.31	0.1	0	0	0.8454
cycloheptane	0.35	0.1	0	0	0.9863
cyclooctane	0.41	0.1	0	0	1.1272
cyclohexene	0.4	0.2	0	0.1	0.8024
cyclohexadiene	N/A	N/A	N/A	N/A	N/A
benzene	0.61	0.52	0	0.14	0.7164
toluene	0.6	0.52	0	0.14	0.8573
ethylbenzene	0.61	0.51	0	0.15	0.9982
methyl chloride	N/A	N/A	N/A	N/A	N/A
methylene chloride	N/A	N/A	N/A	N/A	N/A

Vapor	E	S	A	B	V
chloroform	0.43	0.49	0.15	0.02	0.6167
carbon tetrachloride	0.46	0.38	0	0	0.7391
butyl chloride	N/A	N/A	N/A	N/A	N/A
pentyl chloride	N/A	N/A	N/A	N/A	N/A
chlorohexane	N/A	N/A	N/A	N/A	N/A
chlorooctane	N/A	N/A	N/A	N/A	N/A
1,1-dichloroethane	0.32	0.49	0.1	0.1	0.6352
1,2-dichloroethane	0.42	0.64	0.1	0.11	0.6352
methylchloroform	N/A	N/A	N/A	N/A	N/A
trichloroethylene	0.52	0.37	0.08	0.03	0.7146
chlorobenzene	0.72	0.65	0	0.07	0.8388
acetone	0.18	0.7	0.04	0.49	0.547
methyl ethyl ketone	0.17	0.7	0	0.51	0.6879
tetrahydrofuran	0.29	0.52	0	0.48	0.6223
dioxane	0.33	0.75	0	0.64	0.681
methyl acetate	0.14	0.64	0	0.45	0.6057
ethyl acetate	0.11	0.62	0	0.45	0.7466
propyl acetate	0.09	0.6	0	0.45	0.8875
n-butyl acetate	0.07	0.6	0	0.45	1.0284
ethanol	0.25	0.42	0.37	0.48	0.4491
1-propanol	0.24	0.42	0.37	0.48	0.59
1-butanol	0.22	0.42	0.37	0.48	0.7309
1-pentanol	0.22	0.42	0.37	0.48	0.8718

S4. UNIFAC + FV and UNIFAC 2.0 + FV Henry Solubility Predictions

The UNIFAC + FV and UNIFAC 2.0 + FV models predict the Henrys solubilities within multiplicative factor of 3.7 and 3.0, respectively, at 95% prediction coverage. The probability densities for both models are reported in Figure S7. In general, for vapor-polymer solubilities that can be predicted by both UNIFAC models, UNIFAC 2.0 with machine-learned interaction parameters outperforms UNIFAC. Further, there were several vapor solubilities that could not be estimated on PDMS by UNIFAC because some group interaction parameters are not yet included in the model. For these vapor-polymer combinations, UNIFAC 2.0 had a 95% prediction coverage of a factor of 1.7. This is a small dataset and should not be treated as UNIFAC 2.0 + FV exhibiting higher performance on out-of-set interaction parameters, but it does demonstrate that these new machine-learned interaction parameters may be useful for prediction vapor-polymer interactions that are currently inaccessible to the UNIFAC + FV model.

The R, Q, and interaction parameters for UNIFAC[2] and UNIFAC 2.0[3] are provided in Table S6–Table S9. The quantity of each functional group in the polymer and vapor structures are reported in Table S10 and Table S11. Densities used for the free volume correction terms are reported in Table S12 and Table S13. Note that three size parameters differed minorly between UNIFAC 2.0 and UNIFAC 1.0, likely because of typographical mistakes in the source material or rounding differences. Methyl chloride and trichloroethylene could not be modeled in either UNIFAC formulation. Several polymers had CHCOO groups that could not be modeled in either UNIFAC formulation. A single H-atom was added to change these groups to CH_2COO groups that could be modelled in both UNIFAC formulations.

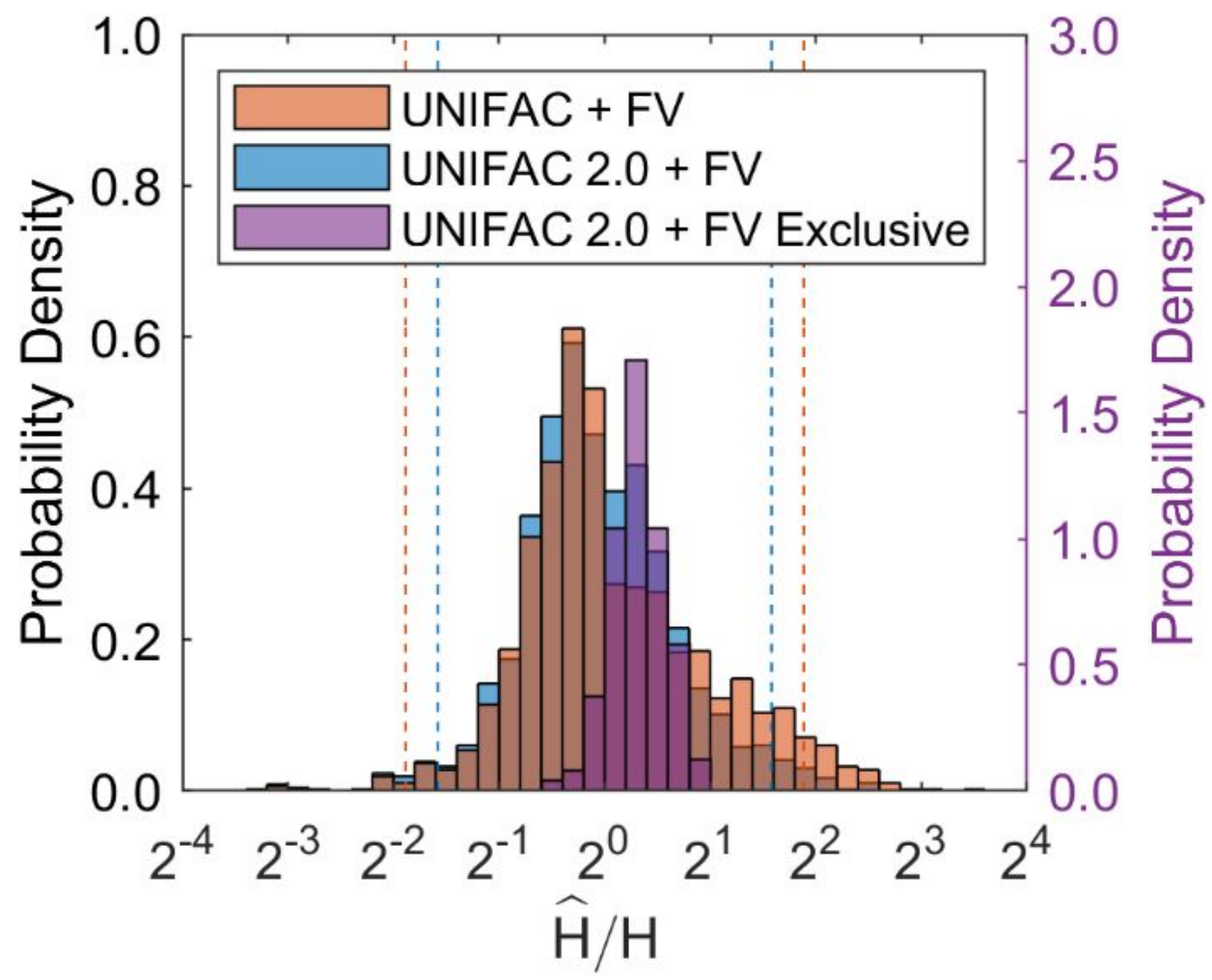

Figure S7. Probability density of the multiplicative residuals for (left axis): UNIFAC + FV, UNIFAC 2.0 + FV, and (right axis) UNIFAC 2.0 + FV Exclusive, where "exclusive" refers to interactions parameters not present in the non-machine learned formulation of UNIFAC. Vertical dashed lines are the 95% prediction intervales for UNIFAC + FV and UNIFAC 2.0 + FV.

Table S6. Size, shape, molar mass, and group information for UNIFAC 1.0 for each functional group.

Subgroup	Q	R	M	Group
CH3	0.848	0.9011	15	1
CH2	0.54	0.6744	14	1
CH	0.228	0.4469	13	1
CH=CH	0.867	1.1167	26	2
ACH	0.4	0.5313	13	3
AC	0.12	0.3652	12	3
OH	1.2	1	17	5
CH3CO	1.448	1.6724	43	9
CH3COO	1.728	1.9031	59	11
CH2COO	1.42	1.6764	58	11
CH2O	0.78	0.9183	30	13

THF	1.1	0.9183	72	13
CH2Cl	1.264	1.4654	49.453	21
CH2Cl2	1.998	2.2564	84.906	22
CHCl2	1.684	2.0606	83.906	22
CHCl3	2.41	2.87	119.359	23
CCl3	2.184	2.6401	118.359	23
CCl4	2.91	3.39	153.812	24
ACCl	0.844	1.1562	47.453	25
SiO	0.466	1.1044	44	43

Table S7. Group interaction parameters in UNIFAC 1.0.

Group	1	2	3	5	9	11	13	21	22	23	24	25	43
1	0	86.02	61.13	986.5	476.4	232.1	251.5	35.93	53.76	24.9	104.3	11.44	252.7
2	-35.36	0	38.81	524.1	182.6	37.85	214.5	-36.87	58.55	-13.99	-109.7	100.1	N/A
3	-11.12	3.446	0	636.1	25.77	5.994	32.14	-18.81	-144.4	-231.9	3	187	238.9
5	156.4	457	89.6	0	84	101.1	28.06	75.62	65.28	-98.12	143.1	123.5	202.7
9	26.76	42.92	140.1	164.5	0	-213.7	-103.6	-191.7	-130.3	-354.6	-39.2	-119.8	N/A
11	114.8	132.1	85.84	245.4	372.2	0	-235.7	-34.74	108.9	-209.7	54.57	442.4	N/A
13	83.36	26.51	52.13	237.7	191.1	461.3	0	301.1	137.8	-154.3	47.67	134.8	N/A
21	91.46	40.25	4.68	562.2	286.3	35.38	225.4	0	108.3	249.2	62.42	153	N/A
22	34.01	-23.5	121.3	527.6	82.86	-132.9	-197.7	-84.53	0	N/A	56.33	223.1	N/A
23	36.7	51.06	288.5	742.1	552.1	176.5	-20.93	-157.1	N/A	0	-30.1	192.1	N/A
24	-78.45	160.9	-4.7	856.3	372	129.5	113.9	11.8	17.97	51.9	0	-75.97	233.1
25	106.8	70.32	-97.27	325.7	518.4	-171.1	-25.15	-129.7	-8.309	-0.2266	-248.4	0	N/A
43	110.2	N/A	234.4	84.85	N/A	N/A	N/A	N/A	N/A	N/A	70.81	N/A	0

Table S8. Group size information in UNIFAC 2.0 that differs from the source used for UNIFAC 1.0. All other size, shape, molar mass, and group parameters are the same as those in Table 6.

Subgroup	Q
CH3CO	1.488
CH2Cl2	1.988
SiO	0.4657

Table S9. Group interaction parameter for UNIFAC 2.0.

Group	1	2	3	5	9	11	13	21	22	23	24	25	43
1	0.00	28.48	11.67	936.82	432.58	273.29	224.37	195.56	151.44	33.24	7.72	84.26	45.65
2	17.00	0.00	26.62	673.86	233.00	148.10	55.79	146.08	102.99	65.77	21.45	103.19	51.89
3	37.20	14.65	0.00	521.82	187.32	105.22	32.99	56.07	23.77	-12.42	12.98	85.34	31.66
5	279.89	323.68	230.49	0.00	10.97	60.69	19.20	232.15	211.65	235.75	284.07	380.66	-58.25
9	70.44	19.10	-43.52	177.84	0.00	-19.82	-66.73	-117.85	-194.58	-173.77	-25.84	55.59	-43.34
11	96.34	57.43	-19.58	195.18	50.68	0.00	-42.40	-54.06	-124.73	-108.29	5.66	78.47	-42.80
13	43.02	11.75	-48.48	230.09	115.15	53.35	0.00	-50.09	-104.70	-132.89	-42.38	-3.63	-29.14
21	-37.09	-65.05	-57.46	431.91	148.02	82.71	-26.43	0.00	-6.78	-38.58	-56.94	-20.40	57.35
22	-19.48	-63.95	-35.86	428.43	92.09	40.77	-70.23	18.66	0.00	-9.82	-33.96	40.79	53.95
23	7.07	-18.92	-14.98	422.08	126.53	82.83	-45.00	57.87	25.91	0.00	-13.00	49.27	46.73
24	16.86	7.14	0.55	764.42	330.28	215.21	120.74	154.28	117.73	31.30	0.00	63.77	57.23
25	-5.02	-26.00	-29.03	378.60	153.28	106.64	-5.62	66.40	48.77	3.27	-32.09	0.00	46.10
43	19.80	22.98	40.07	214.53	56.50	20.23	51.29	55.38	52.87	56.43	48.09	83.41	0.00

Table S10. Number of each functional group used for each of the polymer structures.

Polymer	CH3	CH2	CH	OH	CH2COO	CH2O	CH2Cl	SiO
poly(ethylethylene)	1000	2000	1000	0	0	0	0	0
polypropylene	1000	1000	1000	0	0	0	0	0
poly(dimethylsiloxane)	2000	0	0	0	0	0	0	1000
polycaprolactone	0	4000	0	0	1000	0	0	0
polyepichlorohydrin	0	0	1000	0	0	1000	1000	0
poly(2-hydroxyethyl acrylate)	0	3000	0	1000	1000	0	0	0
poly(methyl acrylate)	1000	1000	0	0	1000	0	0	0
poly(ethyl acrylate)	1000	2000	0	0	1000	0	0	0
poly(n-butyl methacrylate)	2000	4000	0	0	1000	0	0	0

Table S11. Number of each functional group used in each of the vapor structures.

Vapor Name	CH3	CH2	CH=CH	ACH	AC	OH	CH3CO	CH3COO	CH2O	THF	CH2Cl	CH2Cl2	CHCl2	CHCl3	CCl3	CCl4	ACCl
propane	2	1	0	0	0	0	0	0	0	0	0	0	0	0	0	0	0
butane	2	2	0	0	0	0	0	0	0	0	0	0	0	0	0	0	0
pentane	2	3	0	0	0	0	0	0	0	0	0	0	0	0	0	0	0
hexane	2	4	0	0	0	0	0	0	0	0	0	0	0	0	0	0	0
heptane	2	5	0	0	0	0	0	0	0	0	0	0	0	0	0	0	0
octane	2	6	0	0	0	0	0	0	0	0	0	0	0	0	0	0	0
nonane	2	7	0	0	0	0	0	0	0	0	0	0	0	0	0	0	0
decane	2	8	0	0	0	0	0	0	0	0	0	0	0	0	0	0	0
undecane	2	9	0	0	0	0	0	0	0	0	0	0	0	0	0	0	0
cyclopentane	0	5	0	0	0	0	0	0	0	0	0	0	0	0	0	0	0
cyclohexane	0	6	0	0	0	0	0	0	0	0	0	0	0	0	0	0	0
cycloheptane	0	7	0	0	0	0	0	0	0	0	0	0	0	0	0	0	0
cyclooctane	0	8	0	0	0	0	0	0	0	0	0	0	0	0	0	0	0
cyclohexene	0	4	1	0	0	0	0	0	0	0	0	0	0	0	0	0	0
cyclohexadiene	0	2	2	0	0	0	0	0	0	0	0	0	0	0	0	0	0
benzene	0	0	0	6	0	0	0	0	0	0	0	0	0	0	0	0	0
toluene	1	0	0	5	1	0	0	0	0	0	0	0	0	0	0	0	0
ethylbenzene	1	1	0	5	1	0	0	0	0	0	0	0	0	0	0	0	0
methyl chloride	0	0	0	0	0	0	0	0	0	0	0	0	0	0	0	0	0
methylene chloride	0	0	0	0	0	0	0	0	0	0	0	1	0	0	0	0	0
chloroform	0	0	0	0	0	0	0	0	0	0	0	0	0	1	0	0	0
carbon tetrachloride	0	0	0	0	0	0	0	0	0	0	0	0	0	0	0	1	0
butyl chloride	1	2	0	0	0	0	0	0	0	0	1	0	0	0	0	0	0
pentyl chloride	1	3	0	0	0	0	0	0	0	0	1	0	0	0	0	0	0
chlorohexane	1	4	0	0	0	0	0	0	0	0	1	0	0	0	0	0	0
chlorooctane	1	6	0	0	0	0	0	0	0	0	1	0	0	0	0	0	0
1,1-dichloroethane	1	0	0	0	0	0	0	0	0	0	0	0	1	0	0	0	0
1,2-dichloroethane	0	0	0	0	0	0	0	0	0	0	2	0	0	0	0	0	0

Vapor Name	CH3	CH2	CH=CH	ACH	AC	OH	CH3CO	CH3COO	CH2O	THF	CH2Cl	CH2Cl2	CHCl2	CHCl3	CCl3	CCl4	ACCl
methylchloroform	1	0	0	0	0	0	0	0	0	0	0	0	0	0	1	0	0
trichloroethylene	0	0	0	0	0	0	0	0	0	0	0	0	0	0	0	0	0
chlorobenzene	0	0	0	5	0	0	0	0	0	0	0	0	0	0	0	0	1
acetone	1	0	0	0	0	0	1	0	0	0	0	0	0	0	0	0	0
methyl ethyl ketone	1	1	0	0	0	0	1	0	0	0	0	0	0	0	0	0	0
tetrahydrofuran	0	3	0	0	0	0	0	0	0	1	0	0	0	0	0	0	0
dioxane	0	2	0	0	0	0	0	0	2	0	0	0	0	0	0	0	0
methyl acetate	1	0	0	0	0	0	0	1	0	0	0	0	0	0	0	0	0
ethyl acetate	1	1	0	0	0	0	0	1	0	0	0	0	0	0	0	0	0
propyl acetate	1	2	0	0	0	0	0	1	0	0	0	0	0	0	0	0	0
n-butyl acetate	1	3	0	0	0	0	0	1	0	0	0	0	0	0	0	0	0
Ethanol	1	1	0	0	0	1	0	0	0	0	0	0	0	0	0	0	0
1-Propanol	1	2	0	0	0	1	0	0	0	0	0	0	0	0	0	0	0
1-Butanol	1	3	0	0	0	1	0	0	0	0	0	0	0	0	0	0	0
1-Pentanol	1	4	0	0	0	1	0	0	0	0	0	0	0	0	0	0	0

Table S12. Polymer densities used for free volume corrections.

Polymer	density (g/cm3)
poly(ethylethylene)	0.91
polypropylene	0.905
poly(dimethylsiloxane)	0.97
polycaprolactone	1.145
polyepichlorohydrin	1.275
poly(2-hydroxyethyl acrylate)	1.2
poly(methyl acrylate)	1.19
poly(ethyl acrylate)	1.14
poly(n-butyl methacrylate)	1.06

Table S13. Liquid densities of vapors used for free volume corrections.

Vapor	density (g/cm3)
propane	0.5
butane	0.58
pentane	0.626
hexane	0.655
heptane	0.684
octane	0.703
nonane	0.718
decane	0.73
undecane	0.74
cyclopentane	0.745
cyclohexane	0.779
cycloheptane	0.81
cyclooctane	0.83
cyclohexene	0.811
cyclohexadiene	0.84
benzene	0.879
toluene	0.867
ethylbenzene	0.867
methyl chloride	0.92
methylene chloride	1.33
chloroform	1.49
carbon tetrachloride	1.59
butyl chloride	0.886

Vapor	density (g/cm3)
pentyl chloride	0.88
chlorohexane	0.879
chlorooctane	0.87
1,1-dichloroethane	1.18
1,2-dichloroethane	1.25
methylchloroform	1.34
trichloroethylene	1.46
chlorobenzene	1.11
acetone	0.791
methyl ethyl ketone	0.805
tetrahydrofuran	0.889
dioxane	1.03
methyl acetate	0.932
ethyl acetate	0.902
propyl acetate	0.888
n-butyl acetate	0.882
Ethanol	0.789
1-Propanol	0.803
1-Butanol	0.81
1-Pentanol	0.811

References

(1) Helmholtz Centre for Environmental Research-UFZ. UFZ-LSER Database 4.1.1, 2025. https://www.ufz.de/lserd (accessed 2026-07-12).
(2) University of East Anglia. *UNIFAC Structural Groups and Parameters*. Extended AIM Aerosol Thermodynamics Model. https://www.aim.env.uea.ac.uk/aim/info/UNIFACgroups.html (accessed 2026-07-27).
(3) Hayer, N.; Wendel, T.; Mandt, S.; Hasse, H.; Jirasek, F. Advancing Thermodynamic Group-Contribution Methods by Machine Learning: UNIFAC 2.0. *Chem. Eng. J.* **2025**, *504*, 158667. https://doi.org/10.1016/j.cej.2024.158667.
